# Suppressing catalyst poisoning in the carbodiimide-fueled reaction cycle†

**DOI:** 10.1039/d3sc04281b

**Published:** 2023-10-17

**Authors:** Xiaoyao Chen, Héctor Soria-Carrera, Oleksii Zozulia, Job Boekhoven

**Affiliations:** a Department of Chemistry, School of Natural Science, Technical University of Munich Lichtenbergstrasse 4 85748 Garching bei München Germany job.boekhoven@tum.de

## Abstract

In biology, cells regulate the function of molecules using catalytic reaction cycles that convert reagents with high chemical potential (fuel) to waste molecules. Inspired by biology, synthetic analogs of such chemical reaction cycles have been devised, and a widely used catalytic reaction cycle uses carboxylates as catalysts to accelerate the hydration of carbodiimides. The cycle is versatile and easy to use, so it is widely applied to regulate motors, pumps, self-assembly, and phase separation. However, the cycle suffers from side reactions, especially the formation of *N*-acylurea. In catalytic reaction cycles, side reactions are disastrous as they decrease the fuel's efficiency and, more importantly, destroy the molecular machinery or assembling molecules. Therefore, this work tested how to suppress *N*-acylurea by screening precursor concentration, its structure, carbodiimide structure, additives, temperature, and pH. It turned out that the combination of low temperature, low pH, and 10% pyridine as a fraction of the fuel could significantly suppress the *N*-acylurea side product and keep the reaction cycle highly effective to regulate successful assembly. We anticipate that our work will provide guidelines for using carbodiimide-fueled reaction cycles to regulate molecular function and how to choose optimal conditions.

## Abstract 2

*N*-Acylurea, a notorious side product, poisons catalysts in the carbodiimide-fueled reaction cycles. This work tested how to suppress catalyst poisoning, providing guidelines for regulating molecular function and choosing an optimal condition.

## Graphical abstract

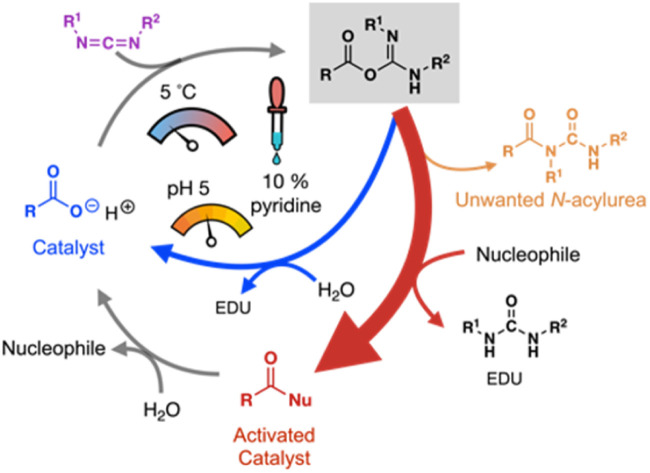

## Introduction

**Scheme 1:**
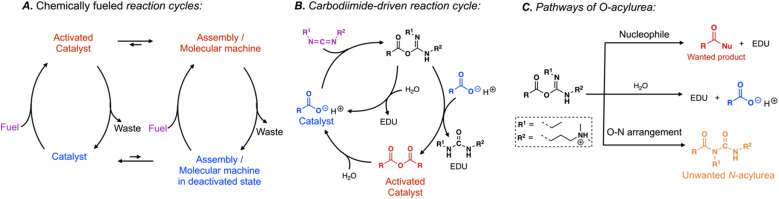
Simplified representation of (A) chemically fueled reaction cycles used to regulate self-assembly or molecular machinery, (B) our carbodiimide-fueled reaction cycle to form anhydrides, and (C) pathways of the intermediate *O*-acylurea.

Chemical reaction networks that catalyze the conversion of a reagent with high chemical potential (fuels) into lower-chemical-potential “waste” molecules are used to power the cells' molecular machinery or molecular assembly. The ATP-driven ATPase pump and GTP-driven dynamic assembly of tubulin into microtubules are prototypical examples.^1–3^ Inspired by such biomolecular machinery, reaction cycles that catalytically convert fuels into waste have been devised to power synthetic molecular machineries like the dynamic assembly of fibers driven by the hydrolysis of methylating agents^4^ or the rotation of a molecular motor catalyzed by the hydration of carbodiimides.^5^ In these reaction cycles, two chemical reactions operate simultaneously: (1) an activation reaction is the reaction between the high chemical potential (which we call fuel) and a catalyst, which activates the catalyst, and (2) a deactivation reaction that reverts the activated catalyst to its original state (Scheme 1A). The free energy released by the two reactions can be used to operate molecular machinery or to regulate self-assembly. For example, suppose the activated catalyst can self-assemble in its short lifetime. In that case, the corresponding assemblies are dynamic because their building blocks are constantly exchanged with freshly activated building blocks. These exchange dynamics result in kinetically controlled assemblies vastly different from their in-equilibrium counterparts. Similarly, if the activated catalyst can undergo a supramolecular interaction, the chemical fuel can power supramolecular machinery like pumps or motors.

Our group and the Hartley group introduced a particularly powerful reaction cycle driven by the hydration of carbodiimide-based fuels (Scheme 1B).^6–8^ In the activation, the carbodiimide fuel activates a carboxylate-containing catalyst, for example, by converting it into its corresponding anhydride. The anhydride spontaneously hydrolyzes in the deactivation. Thus, the carboxylate catalyzes the hydration of the carbodiimide fuel to its corresponding urea waste by transiently becoming an activated catalyst in the form of an anhydride. The carbodiimide-fueled reaction cycle has gained widespread attention. It is frequently used to regulate the phase separation of complex coacervate- and oil-based droplets,^9–12^ the formation of macrocycles,^7,13,14^ the aggregation of nanoparticles,^15^ the formation of vesicles and tubes,^16–18^ the self-assembly of peptides into fibers,^6,19–26^ the crystallization of amino acids,^27^ and the crosslinking of polymer networks.^28–32^ More recently, the cycle was used to drive molecular motors and regulate molecular pumps,^5,33^ and control DNA folding.^34^

Despite its success, the carbodiimide-driven reaction cycle has drawbacks. Most prominently are side reactions that arise from the intermediate *O*-acylurea state—after the reaction between the carbodiimide and the catalyst, this short-lived species is formed^35^ and reacts in three pathways (Scheme 1C). Firstly, it reacts with a nucleophile like a carboxylate to form the desired activated catalyst. Secondly, the *O*-acylurea can hydrolyze by reacting with water which means the catalyst is recovered without performing its function, *i.e.*, inefficient fuel use. Lastly, the *O*-acylurea undergoes an intramolecular O–N arrangement, yielding its corresponding *N*-acylurea.^35–38^ This reaction is irreversible; thus, forming *N*-acylurea decreases the fuel's efficiency and irreversibly destroys the catalyst, *i.e.*, the side reaction poisons the catalyst. The latter reaction is disastrous for chemically fueled reaction cycles. In context, even if only 5% of converted fuel yielded the *N*-acylurea, 99% of the catalyst would be poisoned after it undergoes 100 activation–deactivation cycles (0.95^100^ = 0.59%). The elegance of chemically fueled biomolecular machinery is that it can run thousands of cycles. For synthetic molecular machinery to live up to these levels, catalyst poisoning through side reactions must be understood and suppressed.

Our previous research mainly utilized aspartates and succinates as catalysts, which possess a second carboxylate in proximity, leading to the formation of a 5-membered anhydride.^6,39^ In these reaction cycles, the intramolecular cyclization reaction was rapid, resulting in the absence of *N*-acylurea formation. Additionally, we also investigated reaction cycles involving catalysts with only one carboxylate to form NHS esters, intermolecular anhydrides and oxazolones.^40–43^ In all those reaction cycles, *N*-acylurea is formed. We tried either by using a high fuel-to-catalyst ratio or adding a significant amount of pyridine as an additive to suppress this side product. However, a quantitative understanding or a general rule of the extent to which parameters such as pH, temperature, additives, carbodiimide structures, and catalyst structures can suppress *N*-acylurea has not been established. Therefore, in this work, we tested how simple parameters could be varied to suppress any side reaction in the popular carbodiimide-fueled reaction cycle. The concentration and the structure of the catalyst, the structure of the carbodiimides, the presence of additives, and environmental conditions like temperature and pH were all screened using a kinetic analysis to suppress unwanted *N*-acylurea and increase the yield of the main product. Our work provides guidelines for using carbodiimide-fueled reaction cycles to regulate molecular function by choosing the optimal conditions. We apply these guidelines to a case study and show that the difference between optimal conditions and sub-optimal conditions can be the difference between chemically fueled assemblies or not.

## Results and discussion

**Fig. 1:**
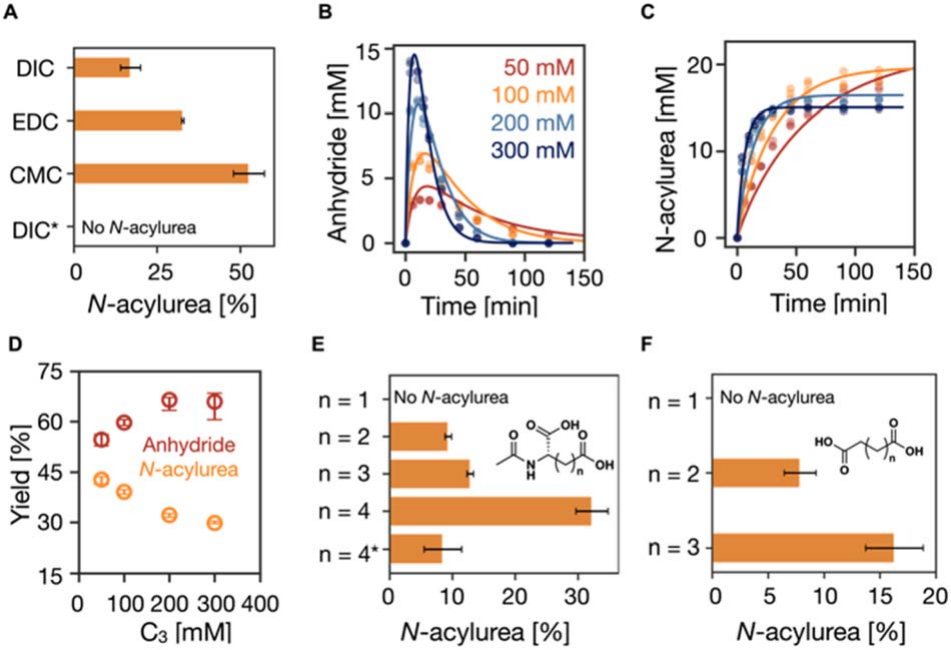
The influence of fuels and catalysts on the suppression of *N*-acylurea. (A) The influence of the structure of fuels on the suppression of *N*-acylurea. The conditions are 75 mM C_3_ in 200 mM MES buffer at pH 6 at 21 °C fueled with 15 mM EDC, CMC, and DIC. * was the reaction cycle conducted at pH 5 at 5 °C with 1.5 mM (10% of the fuel) pyridine. (B–D) The influence of the catalyst concentration. The kinetics profiles of (B) anhydride concentration and (C) *N*-acylurea concentration in the reaction cycles of 50, 100, 150, and 200 mM C_3_ fueled with 50 mM EDC in MES buffer (200 mM, pH 6 at 21 °C). HPLC data (markers) and the kinetic model (lines), *n* = 3. (D) The yields of anhydride and *N*-acylurea as a fraction of fuel against C_3_ concentration. Error bars for yield of anhydride are the 95% confidence interval. (E and F) The influence of the structure of the catalyst on the suppression of *N*-acylurea. Derivates of (E) *N*-acetyl-l-aspartic acid and (F) succinic acid. * was the reaction cycle conducted at pH 5 at 5 °C with 5 mM (10% of the fuel) pyridine.

In our reaction cycle, we used propionic acid (C_3_) as a catalyst because it forms a symmetric anhydride that does not self-assemble. We used the C_3_ in 200 mM MES buffer at pH 6 at 21 °C in all experiments unless stated differently. First, we studied the effect of the fuel on the formation of the *N*-acylurea side product. We initiated the reaction cycle by adding 15 mM EDC, CMC, or DIC to 75 mM C_3_ (EDC = 1-ethyl-3-(3-dimethylaminopropyl)carbodiimide, CMC = *N*-cyclohexyl-*N*-(2-morpholinoethyl)carbodiimide methyl-*p*-toluenesulfonate, DIC = *N*,*N*-diisopropylcarbodiimide). After 24 hours, we measured the amount of catalyst that had disappeared by analytical HPLC. We assume here that the formation of the *N*-acylurea is the only pathway by which the precursor is irreversibly converted. Put differently, the concentration *N*-acylurea is equal to the concentration precursor lost. CMC formed the largest amount of unwanted *N*-acylurea (53%, as expressed in the amount of fuel used to make *N*-acylurea), and EDC ranked second with a value of 33% (Fig. 1A). DIC showed the least formation of *N*-acylurea (17%). Because of DIC's poor water solubility, we focused the rest of this study on EDC.

One pathway to suppressing *N*-acylurea formation is to ensure the *O*-acylurea is as short-lived as possible. The *O*-acylurea lifetime can be decreased by increasing the precursor concentration or adding additives like pyridine.^44^ Increasing the catalyst (C_3_) concentration increased the nucleophile concentration, making *O*-acylurea more vulnerable towards being attacked by a second catalyst and resulting in less *N*-acylurea and higher desired product anhydride. Hence, we first studied the effect of precursor concentration on forming *N*-acylurea by fueling the reaction cycle with 50 mM EDC with varying concentrations of C_3_ (50, 100, 200, and 300 mM). To follow the kinetics of the reaction cycle, we used a benzylamine quenching method developed by our group^45^ to measure the concentration of the product and EDC by converting the anhydride to a stable benzylamide and EDC to a guanidine, respectively. We also used a kinetic model to fit the experimental data of species involved in the reaction cycle every minute by solving a set of ordinary differential equations (see ESI†).^7,46^ The rate constants were determined by the model, and we could calculate the efficiency of the cycle, *i.e.*, the percentage of fuel used to form the transient intermolecular anhydride. We found that, with increasing C_3_ concentration, the EDC was consumed faster (Table S2, Fig. S2 and S11†), yielding a higher maximum anhydride concentration (3 mM to 14 mM) which was present for a shorter period (Fig. 1B). Most importantly, the *N*-acylurea showed only a moderate decrease from 20 mM to 15 mM, respectively (Fig. 1B and C). Since the anhydride is a transient product whose activation (formation) and deactivation (hydrolysis) occur simultaneously, the fraction of fuel successfully used to make the anhydride cannot be directly calculated by measuring the anhydride concentration. However, we used the kinetic model to calculate this fraction—by integrating the anhydride hydrolysis.^7^ Increasing C_3_ concentration from 50 mM to 300 mM increased the effective yield from 55% to 66%, while the *N*-acylurea yield decreased from 43% to 30% (Fig. 1D).

From these effective yields, we can define a term we call the selectivity (*S*), *i.e.*, how much fuel is used for anhydride production relative to *N*-acylurea production (eqn (1)). *S* scales from +100% to −100%, where −100% implies only *N*-acylurea is formed and +100% implies only anhydride is formed. We are aiming for an *S* of 100%. *S* is slightly different from simply comparing the yields, as *S* is independent of the *O*-acylurea hydrolyzed. Thus, it is not a measure of the cycle's efficiency but rather a description of the selectivity of anhydride over *N*-acylurea.1
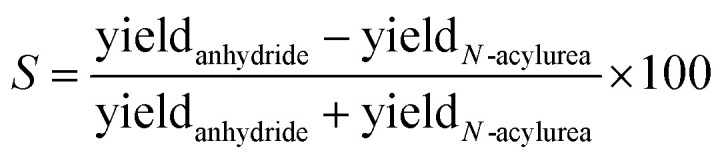


Increasing the C_3_ concentration from 50 mM to 300 mM increased the selectivity from 12% to 38% (Fig. S2Q†). Notably, the effect tended to wear off as the concentration C_3_ increased. Taken together, increasing the C_3_ concentration increases the likelihood of forming the wanted anhydride, but the effect wears off. Besides, decreasing the yield of the *N*-acylurea product to 30% is insufficient for supramolecular machinery—it would imply that less than 1% of the catalyst is present after as little as four cycles.

Given that the *N*-acylurea side product formation decreased with increasing C_3_ concentration, we tested the effect of local concentration effects on the reaction cycle—we used dicarboxylate catalysts that can form intramolecular anhydride in a ring-closing reaction. We evaluated the ring-size effect in aspartate-like (Fig. 1E) and succinate-like derivatives (Fig. 1F) and varied the side chain length from 1 carbon to 4 carbons. We fueled 100 mM dicarboxylate catalysts with 50 mM EDC and determined the lost catalyst concentration to measure the *N*-acylurea concentration formed after 24 hours. No catalyst was lost for *N*-acetyl-l-aspartate (*n* = 1, 5- membered anhydride, Fig. 1E), and HPLC showed no new *N*-acylurea peak. As the neighboring carboxylates were spaced farther from one another by increasing the number of carbons, an increasing concentration of *N*-acylurea was formed. For example, when the side chain has two carbons (*n* = 2, *N*-acetyl-l-glutamate, corresponding to a 6-membered ring anhydride), 9% of the catalyst is lost. This value rises to 32% when the side chain has four carbons (*n* = 4). Similar effects were observed for the succinic acid series (*n* = 1, 2, and 3, Fig. 1F). Taken together, as the distance between the *O*-acylurea and the second carboxylate as a nucleophile increases, the local concentration effect is suppressed, leading to more unwanted side product.

**Fig. 2:**
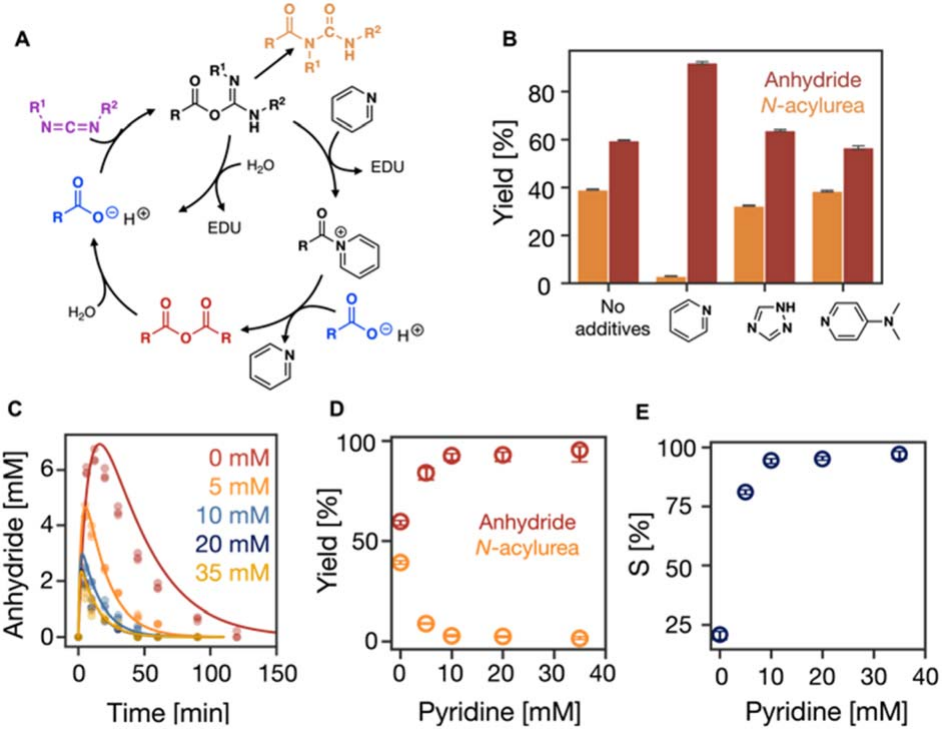
The influence of additives on the suppression of *N*-acylurea. (A) Simplified representation of the reaction cycle with additives, pyridine as an example. (B) The efficiency of the reaction when additives like pyridine, 1,2,4-triazole, and DMAP were used. (C) The kinetic profiles of anhydride concentration in the reaction cycles of 100 mM C_3_ fueled with 50 mM EDC with 0, 5, 10, 20, 35 mM pyridine in MES buffer (200 mM, pH 6 at 21 °C). HPLC data (markers) and the kinetic model (lines), *n* = 3. (D) The yields of anhydride and *N*-acylurea as a fraction of fuel against the pyridine concentration. Error bars for yield of anhydride are the 95% confidence interval. (E) Selectivity as defined in eqn (1) against the pyridine concentrations.

Next, we tested the role of additives in suppressing the unwanted *N*-acylurea. We focused on trapping agents, *i.e.*, reagents like pyridine, that react rapidly with the *O*-acylurea to form intermediates that cannot rearrange to the *N*-acylurea (Fig. 2A).^47,48^ Besides pyridine, we tested the performance of 1,2,4-triazole, and DMAP (4-dimethylaminopyridine), by fueling 100 mM C_3_ with 50 mM EDC with 10 mM of the additives. Compared to the reaction cycle without additives, 1,2,4-triazole and DMAP showed a very limited ability to increase the efficiency and selectivity of the reaction cycle with similar yields of *N*-acylurea (40%) and anhydride (60%) (Fig. 2B, S3 and S12†). In contrast, pyridine significantly suppressed *N*-acylurea formation to 5% of the EDC added, while 92% of EDC was used to form anhydride (Fig. 2B and S3†).

In terms of the mechanism of action, we studied how additives modify the kinetic constants of the reaction cycle. According to our hypothesis, reactions involving *O*-acylurea as a reagent will be significantly modified. In the case of pyridine, anhydride formation (*k*_2_) and *O*-acylurea hydrolysis (*k*_3_) were one order of magnitude faster compared to the without or other additives (Table S3, Fig. S3†). All additives favor anhydride hydrolysis (*k*_4_), while pyridine accelerated hydrolysis most, 12-fold faster than without pyridine, thereby shortening the anhydride half-life from 9 minutes to 46 seconds. Moreover, the decreased half-life also decreased the maximum anhydride formed to 2.5 mM (Table S3, Fig. S3†). Although all additives reduced the half-life of the anhydride, 1,2,4-triazole showed the least catalytic activity and did not significantly decrease the maximum anhydride concentration (Table S3, Fig. S3†). These differences in catalytic activity can be explained by their nucleophilicities in water at pH 6. Even though DMAP has a greater nucleophilicity than pyridine,^49^ its nucleophilicity is decreased because it is protonated at pH 6. DMAP has a higher p*K*_a_ (9.7)^50^ in water than 1,2,4-triazole (p*K*_a_ = 2.4)^51^ and pyridine (p*K*_a_ = 5.23).^52^ Therefore, at pH 6, 1,2,4-triazole and pyridine are not pronated, but 1,2,4-triazole showed the least catalytical ability, because azoles are less nucleophilic than pyridines.^53^

As we established that pyridine is the most successful additive, we tested the effect of its concentration by fueling 100 mM C_3_ with 50 mM EDC with various amounts of pyridine on the selectivity. With increasing pyridine concentration from 0 to 35 mM, the reaction cycle became faster with accelerated rate constants of all reactions. Especially, EDC consumption was faster (Table S4, Fig. S4 and S13†), and anhydride was present for as short as 50 minutes (Fig. 2C). Between a pyridine concentration of 0 and 35 mM, the maximum anhydride decreased from 6.5 mM to 1.6 mM (Fig. 2C) due to the accelerated hydrolysis. However, it remained roughly constant above 10 mM pyridine. Similarly, the amount of EDC used to form the unwanted *N*-acylurea significantly decreased from 39% to 1% but only marginally decreased beyond 10 mM pyridine (Fig. 2D and S4†). Therefore, pyridine concentrations greater than 10 mM result in similar values in the consumption of EDC to produce both anhydride and *N*-acylurea (Fig. 2D, Table S4†). These optimized conditions greatly improve over the original conditions.

In our kinetic model, we do not consider the formation of the acylpyridinium ion formed due to the reaction between *O*-acylurea and pyridine. Hence, all constants related to *O*-acylurea also consider the acylpyridinium ion, which is why they monotonically change with pyridine concentration (Table S4, Fig. S4†). *S* was 20% without pyridine and increased to 97% with 35 mM pyridine, indicating the *N*-acylurea gets much less favored with increasing pyridine. Still, no significant increase was observed between 10 mM pyridine and 35 mM pyridine (Fig. 2E), suggesting the effect of pyridine concentration wears off.

**Fig. 3:**
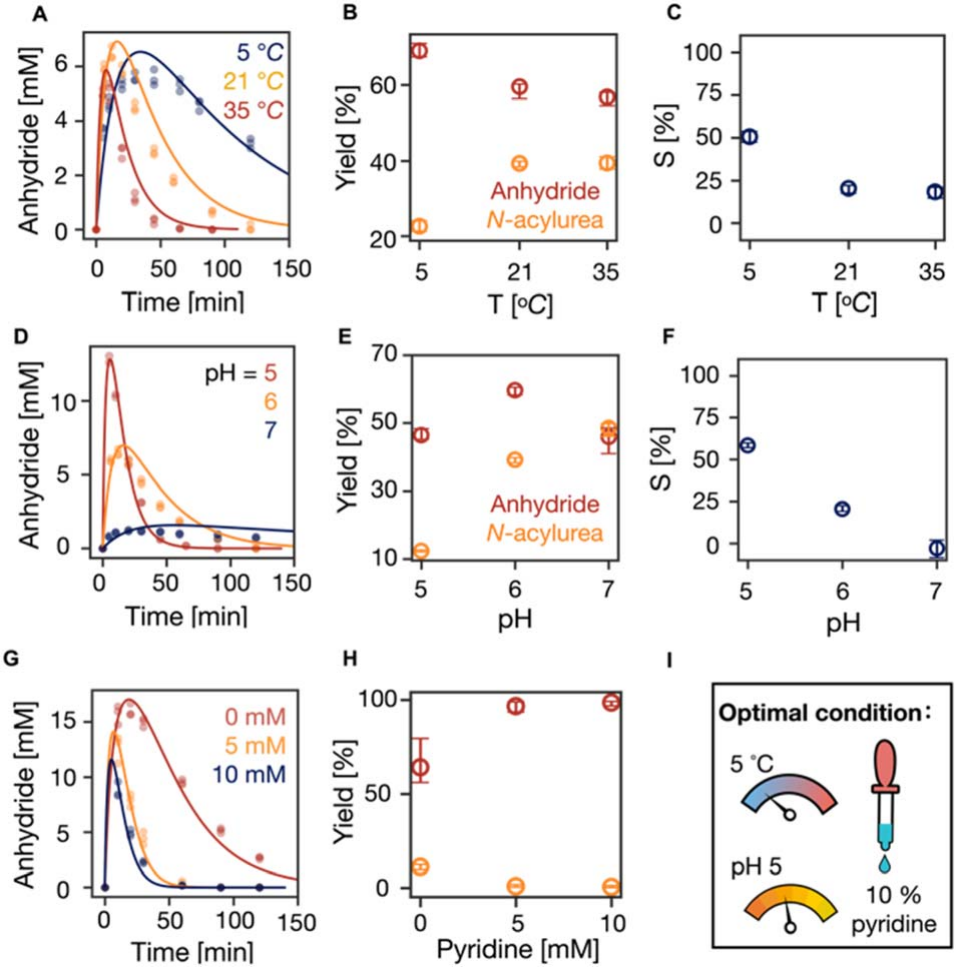
The influence of temperature and pH on the suppression of *N*-acylurea. (A, D and G) The kinetics profiles of anhydride concentration in the reaction cycles of 100 mM C_3_ that were fueled with 50 mM EDC in MES buffer (200 mM) (A) at pH 6 under 5, 21 and 35 °C without pyridine, (D) at pH 5, 6 and 7 at 21 °C without pyridine, (G) at pH 5 at 5 °C with 0, 5, 10 mM pyridine. HPLC data (markers) and the kinetic model (lines), *n* = 3. (B, E and H) The yields of anhydride and *N*-acylure as a fuel fraction under different conditions above. Error bars for yield of anhydride are the 95% confidence interval. (C and F) Selectivity as defined in eqn (1) against temperature and pH, respectively. (I) The optimal condition of our reaction cycle: low temperature at 5 °C, low pH at 5, and the addition of 10% pyridine as a fraction of fuel.

Next, we tested the effect of temperature on the suppression of *N*-acylurea by fueling 100 mM C_3_ with 50 mM EDC at 5, 21, and 35 °C. An increase in temperature tends to increase reaction rates while decreasing selectivity. Indeed, with increasing temperature, the overall reaction cycle became faster (Fig. 3A, S5 and S14, Table S5†). The anhydride concentration peaked at around 5 mM at all the temperatures, but the half-lives of the anhydride decreased from 16 min (5 °C) to 4 min (35 °C), respectively. Moreover, the concentration of *N*-acylurea doubled from 10 mM (5 °C) to 20 mM (35 °C) when increasing the temperature (Fig. S5†). From the kinetic model, it became clear that the amount of EDC used to make anhydride decreased slightly while the fraction of EDC used for *N*-acylurea increased significantly (Fig. 3B). We calculated the selectivity factor, which increased from 20% (21 °C) to 50% (5 °C) and a marginal decrease to 18% (35 °C) (Fig. 3C). We conclude that the formation of the unwanted *N*-acylurea can be suppressed at lower temperatures.

Similarly, we tested the effect of pH on the suppression of *N*-acylurea by fueling 100 mM C_3_ with 50 mM EDC in 200 mM MES buffer at 21 °C at pH 5, 6, and 7. With increasing pH, the overall reaction cycle became much slower because the rate of EDC consumption dropped by orders of magnitude (Table S5, Fig. S6 and S15†). Moreover, with increasing pH from 5 to 7, the anhydride hydrolysis decreased, resulting in an increased anhydride half-life from 7.5 to 19.2 min (Table S5,†Fig. 3D). Due to the decreased reactivity of EDC, the maximum anhydride concentration dropped drastically (Fig. 3D). Nevertheless, the amount of EDC used to produce anhydride varied minimally, with similar values for pH 5 and 7, around 46%, and a slight increase to 60% at pH 6 (Fig. 3E). However, the formed *N*-acylurea concentration increased drastically from pH 5 to 7 (Fig. S6† and3E). In line with these data, the selectivity decreased from 58% to −3% with increasing pH (Fig. 3F), which implies that at higher pH, more EDC is used to make the unwanted *N*-acylurea than the wanted anhydride. Overall, pH 5 showed the best ability to suppress *N*-acylurea and keep the reaction cycle efficient.

We realized from all these data that lower temperature and pH lead to an efficient reaction cycle. We further sought optimized conditions by running the cycle under these conditions at pH 5, 5 °C with 0, 5, and 10 mM pyridine, decreasing the maximum anhydride concentration (Fig. 3G, S7 and S16, Table S6†). At the same time, it suppressed *N*-acylurea (Fig. S7†). The effectiveness of suppressing *N*-acylurea leveled off when the pyridine was more than 5 mM which is 10% of the fuel (Fig. 3H). Indeed, the selectivity significantly increased from 71% (0 mM pyridine) to 98% (10 mM pyridine) (Fig. S7Q†).

Therefore, we conclude that the optimal condition for our reaction cycle is to use dicarboxylic acids as catalysts that lead to 5-membered anhydrides like aspartic acid or succinic acid derivatives. When other catalysts are used, we suggest using 10% pyridine as a fraction of the fuel at pH 5 at 5 °C. Under those optimal conditions, *N*-acylurea was completely suppressed in the cycle of C_3_ with DIC (Fig. 1A and S7, Table S6 and S17†). Moreover, the reaction cycle of the *N*-acetyl-l-aspartate derivate with a 4-carbon side chain (*n* = 4) under our optimal condition resulted in a 4-times decrease in the yield of *N*-acylurea side product (Fig. 1E). Furthermore, we conducted refueling experiments of 100 mM C_3_ with 50 mM EDC under the optimal condition of pH 5 at 5 °C with 5 mM pyridine (10% of the fuel) for 10 times (Fig. S10A†). The yield of the unwanted *N*-acylurea in each refueling experiment stays constant and only 4.2 mM *N*-acylurea accumulated after 10 times of refueling. With our kinetic model, we simulated that the catalyst can now undergo over 142 times of refueling before its concentration falls below 1% (Fig. S10B†). In stark contrast, the catalyst was depleted after 5 times of refueling under the standard condition (pH 6, 21 °C without pyridine) (Fig. S10C†).

**Fig. 4:**
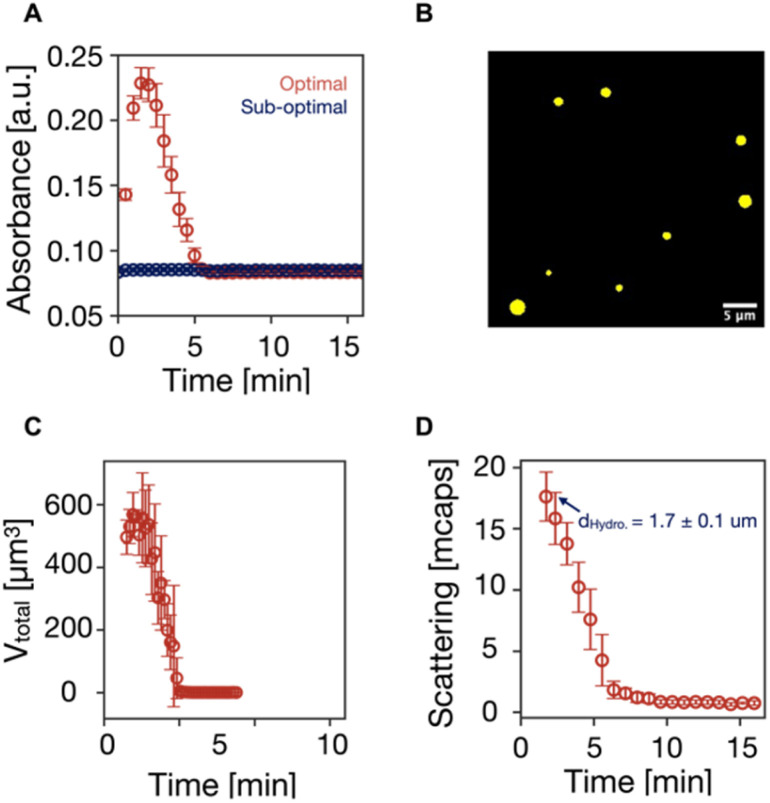
Assembly obtained under the optimal condition. (A) Absorbance at 600 nm by UV/vis spectroscopy as a measure of turbidity of the reaction cycles of 100 mM C_4_ fueled with 100 mM EDC in 200 mM MES buffer at 21 °C under sub-optimal (pH 6 without pyridine) and optimal (pH 5 with 10 mM pyridine) conditions. (B) Confocal fluorescence microscopy image of the oil droplets obtained in the reaction cycle under the optimal condition described in (A). (C) The total volume of oil droplets as a function of time was obtained from confocal fluorescence microscopy. (D) Scattering rate of the droplets as a function of time.

We tested our newly optimized condition in a case using a catalyst that could phase-separate, *i.e.*, butyric acid (C_4_). Due to the extra methylene group compared to C_3,_ the activated catalyst of C_4_ phase separates to form oil droplets. We ran reaction cycles by fueling 100 mM C_4_ with 100 mM EDC under the sub-optimal condition (pH 6 without pyridine) and optimal condition (pH 5 with 10% pyridine as a fraction of EDC). Both reaction cycles were run at 21 °C to compare the phase separation better because phase separation is strongly temperature-dependent.^54^ The reaction cycle under the sub-optimal condition did not turn turbid, suggesting no assembly was observed. However, under the optimal condition, the reaction solution became turbid within 30 seconds after EDC was applied, and this turbidity persisted for 5 minutes, as observed on UV/vis spectroscopy (Fig. 4A). Confocal fluorescence microscopy confirmed that the transient assembly consisted of oil droplets (Fig. 4B). The total volume of the droplets calculated from the confocal fluorescence microscopy peaked at 579 ± 70 μm around 2 min after EDC was added and decreased to 0 after 5 minutes (Fig. 4C). Moreover, the hydrodynamic diameter of the droplets was 1.7 ± 0.1 μm on dynamic light scattering (DLS) (Fig. 4D). The scattering intensity profile further confirmed that droplets emerged upon the application of EDC and decayed after EDC was depleted. The droplets' lifetime was also consistent with the values obtained on UV/vis spectroscopy and confocal fluorescence microscopy. Taken together, using the optimal *versus* sub-optimal conditions can make the difference between obtaining chemically fueled functions or not.

## Conclusions

The carbodiimide-driven reaction cycle has proven a powerful catalytic reaction in powering supramolecular machinery, self-assembly, and phase separation. Nevertheless, side reactions poison the catalyst, which is disastrous for these reaction cycles. We tested the effects of various parameters on suppressing side product *N*-acylurea in a carbodiimide-driven reaction cycle, including fuel structure, catalyst concentration, catalyst structure, additives, temperature, and pH. Increasing catalyst concentration results in less *N*-acylurea formation, but the influence wore off. Catalysts with two carboxylic groups tend to form intramolecular anhydrides with less *N*-acylurea. However, *N*-acylurea formation becomes more favored if the two carboxylic groups are far apart. Moreover, pyridine performed best, but its ability also wore off with increasing concentration. Low temperature and low pH gave a higher effective reaction cycle. Hence, a combination of low temperature, low pH, and 10% pyridine as a fuel fraction is the optimal condition for a highly effective carbodiimide-driven cycle. We believe our work would provide helpful suggestions for using carbodiimide-fueled reaction cycles to regulate molecular function about how to choose an optimal condition.

## Data availability

The data that support the findings of this study are available from the corresponding author upon reasonable request.

## Author contributions

J. B., X. C. O. Z. conceived the research. J. B. and X. C. designed the experiments, analyzed the data, and wrote the manuscript. H. S. C. wrote the kinetic model, fitted all the data, and corrected the manuscript. O. Z. did synthesis.

## Conflicts of interest

The authors declare no competing financial interest.

## Supplementary Material

SC-014-D3SC04281B-s001

## References

[cit1] Moller J. V., Nissen P., Sorensen T. L., Maire M. l. (2005). Transport mechanism of the sarcoplasmic reticulum Ca2+ -ATPase pump. Curr. Opin. Struct. Biol..

[cit2] Toyoshima C. (2008). Structural aspects of ion pumping by Ca2+-ATPase of sarcoplasmic reticulum. Arch. Biochem. Biophys..

[cit3] Brouhard G. J., Rice L. M. (2018). Microtubule dynamics: an interplay of biochemistry and mechanics. Nat. Rev. Mol. Cell Biol..

[cit4] Boekhoven J., Hendriksen W. E., Koper G. J. M., Eelkema R., van Esch J. H. (2015). Transient assembly of active materials fueled by a chemical reaction. Science.

[cit5] Borsley S., Kreidt E., Leigh D. A., Roberts B. M. W. (2022). Autonomous fuelled directional rotation about a covalent single bond. Nature.

[cit6] Tena-Solsona M., Riess B., Grotsch R. K., Lohrer F. C., Wanzke C., Kasdorf B., Bausch A. R., Muller-Buschbaum P., Lieleg O., Boekhoven J. (2017). Non-equilibrium dissipative supramolecular materials with a tunable lifetime. Nat. Commun..

[cit7] Kariyawasam L. S., Hartley C. S. (2017). Dissipative Assembly of Aqueous Carboxylic Acid Anhydrides Fueled by Carbodiimides. J. Am. Chem. Soc..

[cit8] Chen X., Wurbser M. A., Boekhoven J. (2023). Chemically Fueled Supramolecular Materials. Acc. Mater. Res..

[cit9] Donau C., Spath F., Sosson M., Kriebisch B. A. K., Schnitter F., Tena-Solsona M., Kang H. S., Salibi E., Sattler M., Mutschler H., Boekhoven J. (2020). Active coacervate droplets as a model for membraneless organelles and protocells. Nat. Commun..

[cit10] Spath F., Donau C., Bergmann A. M., Kranzlein M., Synatschke C. V., Rieger B., Boekhoven J. (2021). Molecular Design of Chemically Fueled Peptide-Polyelectrolyte Coacervate-Based Assemblies. J. Am. Chem. Soc..

[cit11] Donau C., Spath F., Stasi M., Bergmann A. M., Boekhoven J. (2022). Phase Transitions in Chemically Fueled, Multiphase Complex Coacervate Droplets. Angew. Chem., Int. Ed..

[cit12] Niebuur B. J., Hegels H., Tena-Solsona M., Schwarz P. S., Boekhoven J., Papadakis C. M. (2021). Droplet Formation by Chemically Fueled Self-Assembly: The Role of Precursor Hydrophobicity. J. Phys. Chem. B.

[cit13] Hossain M. M., Atkinson J. L., Hartley C. S. (2020). Dissipative Assembly of Macrocycles Comprising Multiple Transient Bonds. Angew. Chem., Int. Ed..

[cit14] Hossain M. M., Jayalath I. M., Baral R., Hartley C. S. (2022). Carbodiimide-Induced Formation of Transient Polyether Cages**. ChemSystemsChem.

[cit15] Grotsch R. K., Wanzke C., Speckbacher M., Angi A., Rieger B., Boekhoven J. (2019). Pathway Dependence in the Fuel-Driven Dissipative Self-Assembly of Nanoparticles. J. Am. Chem. Soc..

[cit16] Wanzke C., Jussupow A., Kohler F., Dietz H., Kaila V. R. I., Boekhoven J. (2019). Dynamic Vesicles Formed By Dissipative Self-Assembly. ChemSystemsChem.

[cit17] Englert A., Vogel J. F., Bergner T., Loske J., von Delius M. (2022). A Ribonucleotide <?> Phosphoramidate Reaction Network Optimized by Computer-Aided Design. J. Am. Chem. Soc..

[cit18] Sun J., Vogel J., Chen L., Schleper A. L., Bergner T., Kuehne A. J. C., von Delius M. (2022). Carbodiimide-Driven Dimerization and Self-Assembly of Artificial, Ribose-Based Amphiphiles. Chem. –Eur. J..

[cit19] Dai K., Fores J. R., Wanzke C., Winkeljann B., Bergmann A. M., Lieleg O., Boekhoven J. (2020). Regulating Chemically Fueled Peptide Assemblies by Molecular Design. J. Am. Chem. Soc..

[cit20] Kriebisch B. A. K., Jussupow A., Bergmann A. M., Kohler F., Dietz H., Kaila V. R. I., Boekhoven J. (2020). Reciprocal Coupling in Chemically Fueled Assembly: A Reaction Cycle Regulates Self-Assembly and Vice Versa. J. Am. Chem. Soc..

[cit21] Panja S., Adams D. J. (2022). Chemical crosslinking in 'reactive' multicomponent gels. Chem. Commun..

[cit22] Panja S., Dietrich B., Adams D. J. (2019). Chemically Fuelled Self-Regulating Gel-to-Gel Transition. ChemSystemsChem.

[cit23] Yao Z. F., Kuang Y., Kohl P., Li Y., Ardoña H. A. M. (2023). Carbodiimide-Fueled Assembly of π-Conjugated Peptides Regulated by Electrostatic Interactions. ChemSystemsChem.

[cit24] Bal S., Das K., Ahmed S., Das D. (2019). Chemically Fueled Dissipative Self-Assembly that Exploits Cooperative Catalysis. Angew. Chem., Int. Ed..

[cit25] Mondal S., Haldar D. (2021). A transient non-covalent hydrogel by a supramolecular gelator with dynamic covalent bonds. New J. Chem..

[cit26] Chen X., Kriebisch B. A. K., Bergmann A. M., Boekhoven J. (2023). Design rules for reciprocal coupling in chemically fueled assembly. Chem. Sci..

[cit27] Schnitter F., Riess B., Jandl C., Boekhoven J. (2022). Memory, switches, and an OR-port through bistability in chemically fueled crystals. Nat. Commun..

[cit28] Würbser M. A., Schwarz P., Heckel J., Bergmann A. M., Walther A., Boekhoven J. (2021). Chemically Fueled Block Copolymer Self-Assembly into Transient Nanoreactors. ChemSystemsChem.

[cit29] Rajawasam C. W. H., Tran C., Weeks M., McCoy K. S., Ross-Shannon R., Dodo O. J., Sparks J. L., Hartley C. S., Konkolewicz D. (2023). Chemically Fueled Reinforcement of Polymer Hydrogels. J. Am. Chem. Soc..

[cit30] Zhang B., Jayalath I. M., Ke J., Sparks J. L., Hartley C. S., Konkolewicz D. (2019). Chemically fueled covalent crosslinking of polymer materials. Chem. Commun..

[cit31] Heckel J., Loescher S., Mathers R. T., Walther A. (2021). Chemically Fueled Volume Phase Transition of Polyacid Microgels. Angew. Chem., Int. Ed..

[cit32] Lang X., Thumu U., Yuan L., Zheng C., Zhang H., He L., Zhao H., Zhao C. (2021). Chemical fuel-driven transient polymeric micelle nanoreactors toward reversible trapping and reaction acceleration. Chem. Commun..

[cit33] Borsley S., Leigh D. A., Roberts B. M. W., Vitorica-Yrezabal I. J. (2022). Tuning the Force, Speed, and Efficiency of an Autonomous Chemically Fueled Information Ratchet. J. Am. Chem. Soc..

[cit34] Stasi M., Monferrer A., Babl L., Wunnava S., Dirscherl C. F., Braun D., Schwille P., Dietz H., Boekhoven J. (2022). Regulating DNA-Hybridization Using a Chemically Fueled Reaction Cycle. J. Am. Chem. Soc..

[cit35] Kurzer F., Douraghi-Zadeh K. (1967). Advances in the chemistry of carbodiimides. Chem. Rev..

[cit36] DeTar D. F., Silverstein R. (1966). Reactions of Carbodiimides. I. The Mechanisms of the Reactions of Acetic Acid with Dicyclohexylcarbodiimide1,2. J. Am. Chem. Soc..

[cit37] DeTar D. F., Silverstein R. (1966). Reactions of Carbodiimides. II. The Reactions of Dicyclohexylcarbodiimide with Carboxylic Acids in the Presence of Amines and Phenols1,2. J. Am. Chem. Soc..

[cit38] DeTar D. F., Silverstein R., Rogers Jr F. F. (1966). Reactions of Carbodiimides. III. The Reactions of Carbodiimides with Peptide Acids1,2. J. Am. Chem. Soc..

[cit39] Schwarz P. S., Laha S., Janssen J., Huss T., Boekhoven J., Weber C. A. (2021). Parasitic behavior in competing chemically fueled reaction cycles. Chem. Sci..

[cit40] Grotsch R. K., Angi A., Mideksa Y. G., Wanzke C., Tena-Solsona M., Feige M. J., Rieger B., Boekhoven J. (2018). Dissipative Self-Assembly of Photoluminescent Silicon Nanocrystals. Angew. Chem., Int. Ed..

[cit41] Tena-Solsona M., Wanzke C., Riess B., Bausch A. R., Boekhoven J. (2018). Self-selection of dissipative assemblies driven by primitive chemical reaction networks. Nat. Commun..

[cit42] Kriebisch C. M. E., Bergmann A. M., Boekhoven J. (2021). Fuel-Driven Dynamic Combinatorial Libraries. J. Am. Chem. Soc..

[cit43] Chen X., Stasi M., Rodon-Fores J., Grossmann P. F., Bergmann A. M., Dai K., Tena-Solsona M., Rieger B., Boekhoven J. (2023). A Carbodiimide-Fueled Reaction Cycle That Forms Transient 5(4H)-Oxazolones. J. Am. Chem. Soc..

[cit44] Denmark S. E., Beutner G. L. (2008). Lewis base catalysis in organic synthesis. Angew. Chem., Int. Ed..

[cit45] Schnitter F., Boekhoven J. (2020). A Method to Quench Carbodiimide-Fueled Self-Assembly. ChemSystemsChem.

[cit46] Kariyawasam L. S., Kron J. C., Jiang R., Sommer A. J., Hartley C. S. (2020). Structure-Property Effects in the Generation of Transient Aqueous Benzoic Acid Anhydrides by Carbodiimide Fuels. J. Org. Chem..

[cit47] De Rycke N., Couty F., David O. R. (2011). Increasing the reactivity of nitrogen catalysts. Chem. –Eur. J..

[cit48] Lutz V., Glatthaar J., Wurtele C., Serafin M., Hausmann H., Schreiner P. R. (2009). Structural analyses of N-acetylated 4-(dimethylamino)pyridine (DMAP) salts. Chem. –Eur. J..

[cit49] Brotzel F., Kempf B., Singer T., Zipse H., Mayr H. (2007). Nucleophilicities and carbon basicities of pyridines. Chem. –Eur. J..

[cit50] Fulga T., Onciu M., Chiriac C. I. (2003). Syntheses of esters from carboxylic acids and diphenyl carbonate-4-dimethylaminopyridine at room temperature. Rev. Roum. Chim..

[cit51] Siwen L., Zhen Z., Yuelan Z., Meilin L. (2005). 1H-1,2,4-Triazole: An Effective Solvent for Proton-Conducting Electrolytes. Chem. Mater..

[cit52] Gero A., Markhamm J. J. (1951). Studies on pyridines: I. The basicity of pyridine bases. J. Org. Chem..

[cit53] Baidya M., Brotzel F., Mayr H. (2010). Nucleophilicities and Lewis basicities of imidazoles, benzimidazoles, and benzotriazoles. Org. Biomol. Chem..

[cit54] Eisenberg H., Felsenfeld G. (1967). Studies of the temperature-dependent conformation and phase separation of polyriboadenylic acid solutions at neutral pH. J. Mol. Biol..

