# Supplementary material for: Suppressing catalyst poisoning in the carbodiimide-fueled reaction cycle
Source: Chem Sci. 2023 Oct 17;14(44):12653–60. doi: 10.1039/d3sc04281b (PMC10646924; doi:10.1039/d3sc04281b)
Supplement: SC-014-D3SC04281B-s001 [file SC-014-D3SC04281B-s001.pdf]

Electronic Supplementary Material (ESI) for Chemical Science.
This journal is © The Royal Society of Chemistry 2023

Electronic Supplementary Information for:

Suppressing catalyst poisoning in the carbodiimide-fueled reaction cycle

Xiaoyao Chen,[1] Héctor Soria-Carrera,[1] Oleksii Zozulia,[1] Job Boekhoven[1*]

[1] Department of Chemistry, School of Natural Sciences, Technical University of Munich, Lichtenbergstrasse 4, 85748 Garching, Germany.

MATERIALS

Materials. (S)-2-Aminoadipic Acid, and (S)-2-Aminoheptanedioic acid were purchased from BLDpharm. adipic acid, glutaric acid, succinic acid, N-Acetyl-L-aspartic acid (Ac-D-OH), N-Acetyl-L-glutamic acid (Ac-E-OH), propionic acid, butyric acid, 2-(*N*-morpholino) ethanesulfonic acid (MES) buffer, 1-Ethyl-3-[3-dimethylaminopropyl]carbodiimide hydrochloride (EDC HCl), N, N′-Diisopropylcarbodiimide, benzylamine, trifluoroacetic acid (TFA) were purchased from Sigma-Aldrich and used without any further purification unless otherwise indicated. HPLC grade acetonitrile (ACN), and N N-Dimethylformamide were purchased from VWR.

METHODS

Sample preparation. We prepared stock solutions of the precursors by dissolving the acids in 0.2 M MES buffer, then we adjusted the pH to 5.0, 6.0, or 7.0. Benzylamine stocks were prepared freshly in acetonitrile. EDC stock solutions were prepared by dissolving the EDC powder in MQ water. Reaction cycles were started by adding EDC from the freshly prepared stock solution to the precursor.

Peptide synthesis and purification. (S)-2-aminoadipic acid and (S)-2-aminoheptanedioic acid were acetylated using acetic anhydride under ultrasonic conditions as reported before for numerous amino acids, in particular for Glu and Asp.[1] In brief, 100 mg of each amino acid were suspended in 5 mL of acetic anhydride and sonicated for 10 minutes until complete dissolution. Evaporation and further purification of crude solid on a reversed-phase High-Performance Liquid Chromatography (RP-HPLC), detected at 220 nm. The purified acetylated amino acids were lyophilized (Lyophylle: Alpha LDplus, Christ) and characterized by ^{1}H-NMR spectroscopy, HPLC, and Electron Spray Ionisation – Mass Spectrometry (ESI-MS).

Kinetic model. We used a kinetic model to predict the concentration of the anhydride, EDC and N-acylurea over time. We describe the model in supporting Note. The rate constants we used in this work are given in Supporting Table S2 – S6.

Analysis of the reaction kinetics by HPLC. The kinetics of the chemical reaction cycles were monitored over time by analytical HPLC (ThermoFisher Vanquish Duo UHPLC, a Hypersil Gold 100 x 2.1 mm C18 column (3 µm pore size). We used the quenching method[2] to indirectly determine the concentration of the anhydride product by converting it irreversibly into an amide, which we refer to as benzylamide. Also, EDC reacted with benzylamine and was converted irreversibly into a guanidine. After initiating the reaction cycle by adding EDC to the precursor solution, we took a certain amount of reaction solution to the benzylamine solution at each time point. After several hours, the resulting quenched clear solution was measured via HPLC to determine the benzylamide and guanidine concentration. In this work, the ratio of the reaction solution and benzylamine solution (400 mM) was 1:1. 0.5 µL quenched solution was directly injected without further dilution into HPLC and tracked with a UV/Vis detector at 220 and 254 nm. All compounds involved were separated using a method of a linear gradient of H_2O: ACN, each with 0.1% TFA.

HPLC Methods.

Method 1. for Propionic acid, Adipic Acid, N-acetyl-L-2-aminoadipic acid and N-acetyl-L-2-aminoheptanedioic acid: H_2O/ACN starts from 98/2. ACN keeps at 2 % for the first 5 min, increasing to 25% in 14 min, 1 min increasing to 98 %, followed by 1 min 98 %, 1 min going back to 2%, and finally 3 min 2%.

Method 2. for succinic acid and N-Acetyl-L-aspartic acid (Ac-D-OH): H_2O/ACN starts from 99/1. ACN keeps at 1 % for the first 7 min, increasing to 50% in 2 min, followed by 2 min 50 %, 2 min going back to 1%, and finally 4 min 1%.

Method 3. for N-Acetyl-L-glutamic acid (Ac-E-OH): H_2O/ACN starts from 98/2. ACN keeps at 2 % for the first 5 min, increasing to 15% in 8 min, 11 min climbing to 17 %, 1 min increasing to 98 %, followed by 1 min 98 %, 1min going back to 2%, and finally 3 min 2%.

Method 4. for N-Acetyl-L-glutamic acid (Ac-E-OH): H_2O/ACN starts from 98/2. ACN keeps at 2 % for the first 5 min, increasing to 15% in 8 min, 11 min climbing to 17 %, 1 min increasing to 98 %, followed by 1 min 98 %, 1min going back to 2%, and finally 3 min 2%.

Method 5. for glutaric acid: H_2O/ACN starts from 98/2. ACN keeps at 2 % for the first 7 min, increasing to 98% in 2 min, followed by 2 min 98 %, 2 min going back to 2%, and finally 4 min 2%.

Method 6. for Propionic acid when CMC as a fuel: H_2O/ACN starts from 98/2. ACN keeps at 2 % for the first 5 min, increasing to 25% in 5 min, 10 min increasing to 98 %, followed by 1 min 98 %, 1 min going back to 2%, and finally 3 min 2%.

Method 7. for Propionic acid when DIC as a fuel (quenched): H_2O/ACN starts from 98/2. ACN keeps at 2 % for the first 5 min, increasing to 25% in 14 min, 9 min increasing to 98 %, followed by 2 min 98 %, 2 min going back to 2%, and finally 3 min 2%.

Electron Spray Ionisation – Mass Spectrometry (ESI-MS). We used a Varian 500 MS LC ion trap or an LCQ Fleet Ion Trap Mass Spectrometer (Thermo Scientific) to perform ESI-MS measurements. The samples were diluted in acetonitrile or MQ water before injection into the Mass Spectrometer. All recorded MS data were interpreted using the Thermo Xcalibur Qual Browser 2.2 SP1.48 software or a direct photo by a smartphone.

Dynamic light scattering (DLS). DLS experiments were conducted on Litesizer 500 in the measurement mode of particle size series. The measurements were measured with automatic angle and an equilibration time of 5s. Each measurement consisted of 3 acquisitions with an acquisition time of 10s.

Confocal Fluorescence Microscopy. We used a Leica SP5 confocal microscope with a 63x water immersion objective to image the oil droplets. Samples were prepared as described above with a total reaction volume of 30 µL, including 2.5 µM Nile red as a dye. Samples were deposited on an IBIDI 15 wells chambered coverslip with sterilized glass-bottom No. 1.5H non-coated before exciting them with a 552 nm laser and imaging at 580 - 700 nm. All recorded images were analyzed using the ImageJ 1.52p software (Java 1.80_172 (64-bit)).

NMR spectra. NMR spectra were conducted on Bruker AV400US (400 and 500 MHz). Chemical shifts are reported as δ-values in parts per million (ppm) relative to the deuterated solvent peak: DMSO-d_6 (δH: 2.50). For the denotation of the observed signal multiplicities, the following abbreviations were used: m (multiplet), dd (doublet of doublets), d (doublet), and s (singlet).

Turbidity measurements and lifetime determination. Turbidity measurements were carried out at 21 °C on a Microplate Spectrophotometer (Thermo Scientific Multiskan GO, Thermo Scientific SkanIt Software 6.0.1). Measurements were performed in a non-tissue culture treated 96-well plate (Falcon, flat bottom). Every 30 seconds, the absorbance of the 100µL samples was measured at 600 nm. All experiments were performed three times. The lifetime refers to the time it takes for the absorption to drop under 0.01 a.u. (blank subtraction) after EDC addition.

SUPPORTING NOTE

1. Description of the kinetic model

We wrote a kinetic model in Python that described each reaction involved in the chemical reaction network. The concentrations of each reactant were calculated for every 1 minute in the cycle. The model was used to fit the HPLC data that described the evolution of the concentration of precursors, EDC, anhydride, and N-acylurea over time. The model described 5 chemical reactions: direct hydrolysis of EDC (r_0), the activation of acid (r_1), the formation of intermolecular anhydride (r_2), hydrolysis of O-acylurea (r_3), hydrolysis of anhydride (r_4) and the side product formation of N-acylurea by a rearrangement reaction (N to O shift, r_5).

(0) Reaction 0: direct hydrolysis of EDC (k_0). It has a first-order rate constant that can be obtained from experiments. We used analytic HPLC to determine k_0 under different conditions, and the values can be found in Table S5.

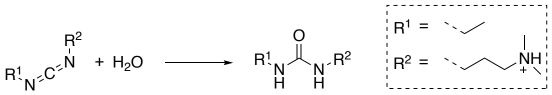

(1) Reaction 1: the activation of acid by EDC to form O-acylurea (k_1). This second-order rate constant was determined for each precursor by HPLC by monitoring the EDC consumption.

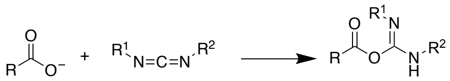

(2) Reaction 2: the formation of intermolecular anhydride (k_2). This second-order rate constant could not be determined because the O-acylurea was not observed.

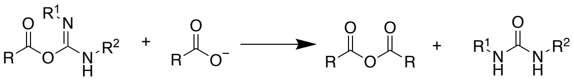

(3) Reaction 3: direct hydrolysis of O-acylurea (k_3). This first-order rate constant could not be obtained because the O-acylurea was not observed.

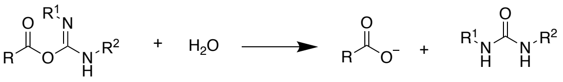

(4) Reaction 4: hydrolysis of anhydride (k_4). As indirectly determined by HPLC using the benzylamine quenching method, the hydrolysis of anhydride takes place with a (pseudo)-first order. The hydrolysis reaction rate was calculated by multiplying the first-order rate constant k_4 with the concentration of anhydride.

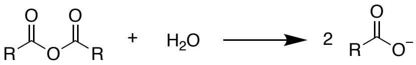

(5) Reaction 5: side product formation of N-acylurea (N to O shift, k_5). This first-order rate constant could not be obtained because the O-acylurea was not observed.

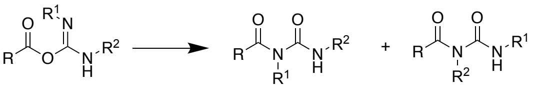

2. Ordinary differential equations (ODEs).

We used the following set of ODEs to describe the systems by using a steady-state approximation that removed any explicit dependence on the concentration of O-acylurea and used an in house developed python model to fit the experimental data.

The following set of ODEs was used and fitted. Ac stands for acid, F for fuel, W for waste, P for product and N for N-acylurea.

$$\begin{cases} \frac{d[Ac]}{dt} = -k_1 \cdot [Ac] \cdot [F] + 2 \cdot k_4 \cdot [P] + \frac{k_3}{k_2 \cdot [Ac] + k_3 + k_5} \cdot k_1 \cdot [Ac] \cdot [F] - \frac{k_2}{k_2 \cdot [Ac] + k_3 + k_5} \cdot k_1 \cdot [Ac]^2 \cdot [F] \\ \frac{d[F]}{dt} = -k_1 \cdot [Ac] \cdot [F] - k_0 \cdot [F] \\ \frac{d[W]}{dt} = +k_0 \cdot [F] + \frac{k_2 \cdot [Ac] + k_3}{k_2 \cdot [Ac] + k_3 + k_5} \cdot k_1 \cdot [Ac] \cdot [F] \\ \frac{d[P]}{dt} = + \frac{k_2}{k_2 \cdot [Ac] + k_3 + k_5} \cdot k_1 \cdot [Ac]^2 \cdot [F] - k_4 \cdot [P] \\ \frac{d[N]}{dt} = + \frac{k_5}{k_2 \cdot [Ac] + k_3 + k_5} \cdot k_1 \cdot [Ac] \cdot [F] \end{cases}$$

3. Python codes.

To fit the data, we used a homemade code inspired by previous works from Hartley's group.[3 4]

The code can be found on GitHub: https://github.com/BoekhovenLab/Catalyst-poisoning

Briefly, the functions of the code are defined in ODE_symmetric_package.

We solve the ODE system and fit it to the experimental data minimizing an error function. We used *lmfit* package (https://zenodo.org/record/11813). We also did bootstrap in the fitting to generate a distribution of kinetic constants that minimized the error. For the fitting, we used the median of the distribution to be more robust towards outliers compared to the mean. We also calculated the root-squared mean error of the fitting to calculate the 95 % confidence interval. We also calculated R^2 to evaluate the goodness of the fitting.

Then we create a function to run the above package before the fitting of the experimental data.

We do another notebook for fitting experimental data. It will first upload the data, then fit and plot it.

Examples of how to fit experimental data are in the GitHub repository.

SUPPORTING TABLES

Supporting Table S1. Mass information of all products in this work.

Name	Structure	Exact Mass (gmol^{-1})	Mass found (gmol^{-1})
Proponic acid-N-acylurea (EDC as a fuel)	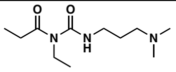 Two possible isomers (we only show one, same as below)	Mw = 229.18<br>$C_{11}H_{23}N_3O_2$	229.94 $[Mw+H^+]$
Proponic acid- benzylamide	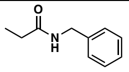	Mw = 163.1<br>$C_{10}H_{13}NO$	163.95 $[Mw+H^+]$
EDC-Guanidine	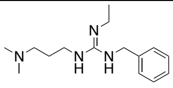	Mw = 262.22<br>$C_{15}H_{26}N_4$	263.11 $[Mw+H^+]$
Ac-E-N-acylurea	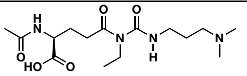	Mw = 344.21<br>$C_{15}H_{28}N_4O_5$	345.02 $[Mw+H^+]$
N-acetyl-L-<br>2-aminoadipic acid	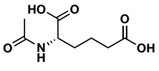	Mw = 203.08<br>$C_8H_{13}NO_5$	203.82 $[Mw+H^+]$<br>647.65 $[3Mw+K^+]$
N-acetyl-L-<br>2-aminoheptanedioic acid	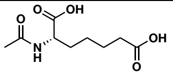	Mw = 217.10<br>$C_9H_{15}NO_5$	217.86 $[Mw+H^+]$<br>456.67 $[2Mw+Na^+]$
N-acetyl-L-<br>2-aminoadipic-N-acylurea<br>and other side products	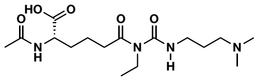<br>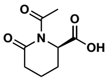<br>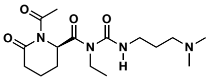	Mw = 358.22<br>$C_{16}H_{30}N_4O_5$<br><br>Mw = 185.07<br>$C_8H_{11}NO_4$<br><br>Mw = 340.21<br>$C_{16}H_{28}N_4O_4$	359.07 $[Mw+H^+]$<br><br>185.76 $[Mw+H^+]$<br>593.69 $[3Mw+K^+]$<br><br>341.05 $[Mw+H^+]$
N-acetyl-L-<br>2-aminoheptanedioic<br>-N-acylurea	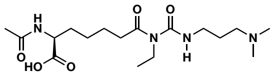	Mw = 372.24<br>$C_{17}H_{32}N_4O_4$	373.09 $[Mw+H^+]$
glutaric-N-acylurea	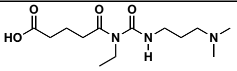	Mw = 287.18<br>$C_{13}H_{25}N_3O_4$	288.04 $[Mw+H^+]$
adipic-N-acylurea	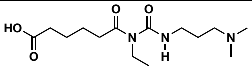<br>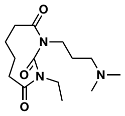	Mw = 301.20<br>$C_{14}H_{27}N_3O_4$<br><br>Mw = 283.19<br>$C_{14}H_{25}N_3O_3$	302.05 $[Mw+H^+]$<br><br>283.92 $[Mw+H^+]$
Propionic acid-CMC-N-acylurea	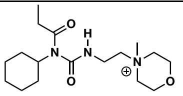	Mw = 326.24<br>$C_{17}H_{32}N_3O_3$	326.06 $[Mw^+]$
Propionic acid-DIC-N-acylurea	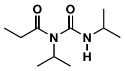	Mw = 200.15<br>$C_{10}H_{20}N_2O_2$	200.84 $[Mw+H^+]$
DIC- Guanidine	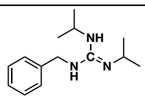	Mw = 233.19<br>$C_{14}H_{23}N_3O_3$	234.16 $[Mw+H^+]$

Supporting Table S2. Rate constants used in the kinetic model for X mM propionic acid with 50 mM EDC without pyridine in MES buffer (200 mM) at pH 6, 21 °C.

	k_0 [min^{-1}]	k_1 [mM^{-1}min^{-1}]	k_2 [mM^{-1}min^{-1}]	k_3 [min^{-1}]	k_4 [min^{-1}]	k_5 [min^{-1}]	Half-life [min][a]	Yield [%][b]
order	1	2	2	1	1	1		
50	1.80×10^{-4}	4.83×10^{-4}	1.59×10^{-1}	1.45×10^{-2}	7.75×10^{-2}	4.28	9.0	55 and 43
100	1.80×10^{-4}	3.97×10^{-4}	9.00×10^{-2}	8.94×10^{-2}	7.65×10^{-2}	4.82	9.1	60 and 39
200	1.80×10^{-4}	4.19×10^{-4}	6.11×10^{-2}	1.55×10^{-1}	9.36×10^{-2}	5.39	7.4	66 and 32
300	1.80×10^{-4}	5.81×10^{-4}	3.93×10^{-2}	7.81×10^{-1}	1.03×10^{-1}	4.95	6.7	66 and 30

[a] Half-life of anhydride [min] (calculated by ln(2)/k_4, [b] Yield of anhydride and N-acylurea as a fraction of the fuel, respectively. Same as below.

Supporting Table S3. Rate constants used in the kinetic model for 100 mM propionic acid with 50 mM EDC with 10 mM additives in MES buffer (200 mM) at pH 6, 21 °C.

	k_0 [min^{-1}]	k_1 [mM^{-1}min^{-1}]	k_2 [mM^{-1}min^{-1}]	k_3 [min^{-1}]	k_4 [min^{-1}]	k_5 [min^{-1}]	Half-life [min][a]	Yield [%][b]
order	1	2	2	1	1	1		
No additives	1.80×10^{-4}	3.97×10^{-4}	9.00×10^{-2}	8.94×10^{-2}	7.65×10^{-2}	4.82	9.1	60 and 39
pyridine	1.80×10^{-4}	7.56×10^{-4}	2.94×10^{-1}	1.58	9.13×10^{-1}	9.54×10^{-1}	0.8	93 and 3
DMAP	1.80×10^{-4}	4.16×10^{-4}	8.21×10^{-2}	1.60×10^{-1}	2.52×10^{-1}	4.66	2.8	57 and 38
triazoloe	1.80×10^{-4}	4.97×10^{-4}	9.74×10^{-2}	3.14×10^{-1}	1.52×10^{-1}	4.18	4.6	64 and 32

[a] [b] see Table S2.

Supporting Table S4. Rate constants used in the kinetic model for 100 mM propionic acid with 50 mM EDC with x mM pyridine in MES buffer (200 mM at pH 6, 21 °C).

	k_0 [min^{-1}]	k_1 [mM^{-1}min^{-1}]	k_2 [mM^{-1}min^{-1}]	k_3 [min^{-1}]	k_4 [min^{-1}]	k_5 [min^{-1}]	Half-life [min][a]	Yield [%][b]
order	1	2	2	1	1	1		
0	1.80×10^{-4}	3.97×10^{-4}	9.00×10^{-2}	8.94×10^{-2}	7.65×10^{-2}	4.82	9.1	60 and 39
5	1.80×10^{-4}	6.52×10^{-4}	1.20×10^{-1}	8.48×10^{-1}	3.79×10^{-1}	1.14	1.8	84 and 9
10	1.80×10^{-4}	7.56×10^{-4}	2.94×10^{-1}	1.58	9.13×10^{-1}	9.54×10^{-1}	0.8	93 and 3
20	1.80×10^{-4}	8.10×10^{-4}	3.92×10^{-1}	1.81	1.25	9.31×10^{-1}	0.6	93 and 2
35	1.80×10^{-4}	7.94×10^{-4}	4.66×10^{-1}	1.92	1.36	9.23×10^{-1}	0.5	95 and 1

[a] [b] see Table S2.

Supporting Table S5. Rate constants used in the kinetic model for 100 mM propionic acid with 50 mM EDC without pyridine in MES buffer (200 mM) at pH x, x °C.

	k_0 [min^{-1}]	k_1 [mM^{-1}min^{-1}]	k_2 [mM^{-1}min^{-1}]	k_3 [min^{-1}]	k_4 [min^{-1}]	k_5 [min^{-1}]	Half-life [min][a]	Yield [%][b]
order	1	2	2	1	1	1		
pH 6, 5 °C	5.43×10^{-5}	1.62×10^{-4}	9.36×10^{-2}	7.38×10^{-1}	4.37×10^{-2}	2.60	15.9	69 and 23
pH 6, 21 °C	1.80×10^{-4}	3.97×10^{-4}	9.00×10^{-2}	8.94×10^{-2}	7.65×10^{-2}	4.82	9.1	60 and 39
pH 6, 35 °C	1.27×10^{-3}	7.54×10^{-4}	8.51×10^{-2}	1.92×10^{-1}	1.77×10^{-1}	4.93	3.9	57 and 39
pH 5, 21 °C	1.58×10^{-3}	3.09×10^{-3}	5.72×10^{-2}	3.63	9.26×10^{-2}	1.15	7.5	47 and 12
pH 7, 21 °C	6.96×10^{-5}	3.48×10^{-5}	6.22×10^{-2}	3.94×10^{-1}	3.61×10^{-2}	5.75	19.2	46 and 48

[a] [b] see Table S2.

Supporting Table S6. Rate constants used in the kinetic model for 100 mM propionic acid with 50 mM EDC with x mM pyridine in MES buffer (200 mM) at pH 5, 5 °C

	k_0 [min^{-1}]	k_1 [$mM^{-1}min^{-1}$]	k_2 [$mM^{-1}min^{-1}$]	k_3 [min^{-1}]	k_4 [min^{-1}]	k_5 [min^{-1}]	Half-life [min][a]	Yield [%][b]
order	1	2	2	1	1	1		
0	1.32×10^{-4}	9.93×10^{-4}	1.78×10^{-1}	5.01	2.87×10^{-2}	2.20	24.1	64 and 11
5	1.32×10^{-4}	1.37×10^{-4}	1.27	8.97×10^{-1}	1.61×10^{-1}	9.13×10^{-1}	4.3	96.6 and 0.96
10	1.32×10^{-4}	1.45×10^{-3}	1.76	1.08	2.54×10^{-1}	9.08×10^{-1}	2.7	98.5 and 0.6
DIC	1.31×10^{-3}	5.23×10^{-3}	4.40×10^{-2}	3.84	5.13×10^{-2}	0	13.5	42 and 0

[a] [b] see Table S2.

Supporting Table S7. Retention time of all the compounds in the quenched reaction cycle.

Precursor \ Retention time / min	precursor	Guanidine	N-acylurea	Benzylamide
Propionic acid (EDC as a fuel)	2.10	14.22	13.43	16.38
Propionic acid (DIC as a fuel)	2.11	22.76	N.D	16.03

Supporting Table S8. Retention time of all the compounds in the reaction cycle without quenching.

Precursor \ Retention time / min	precursor	N-acylurea
Ac-D-OH	1.41	N.D
Ac-E-OH	1.29	10.64
N-acetyl-L-<br>2-aminoadipic acid	1.97	12.73, 16.36<br>8.82 (other side product)
N-acetyl-L-<br>2-aminoheptanedioic acid	5.44	14.42, 15.30
Succinic acid	1.87	N.D
Glutaric acid	2.45	4.096
Adipic Acid	5.51	13.94, 15.23
Propionic acid (CMC as fuel)	2.13	12.68, 14.23

Supporting Table S9. Calibration values of the compounds.

Compound	0.5 uL Calibration value / (a.u.) $(mM)^{-1}$
Propionic acid	0.0294 at 220 nm
Proponic acid-N-acylurea<br>(EDC as a fuel)	3.1892 at 220 nm<br>0.0415 at 254 nm
Benzylamine	0.2099 at 254 nm
Ac-D-OH	0.304 at 220 nm
Ac-E-OH	0.3488 at 220 nm
N-acetyl-L-<br>2-aminoadipic acid	0.3633 at 220 nm
N-acetyl-L-<br>2-aminoheptanedioic acid	0.3836 at 220 nm
Succinic acid	0.050 at 220 nm
Glutaric acid	0.065 at 220 nm
Adipic Acid	0.0766 at 220 nm

SUPPORTING FIGURES

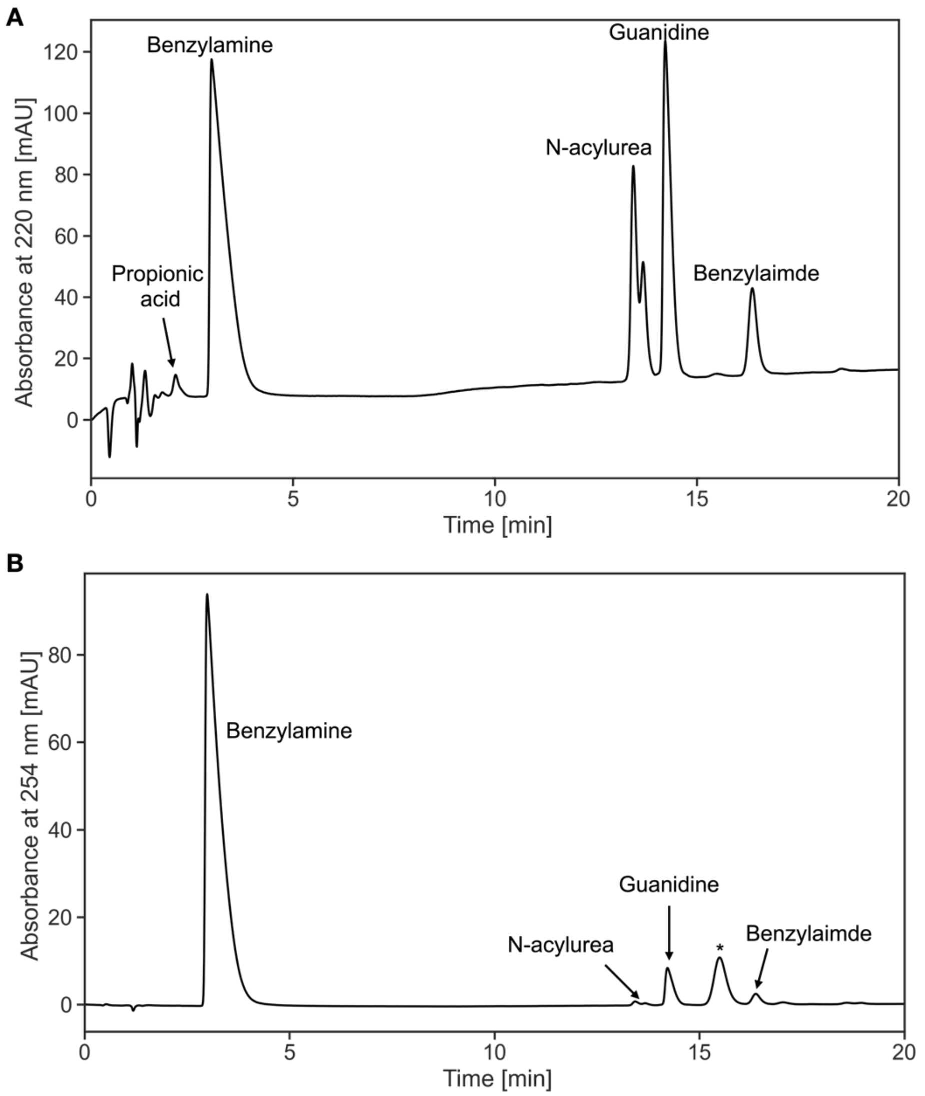

Figure S1. **Examples of HPLC chromatogram of the entire reaction network with benzylamine quenching measured at (A)** 220 nm and **(B)** 254 nm, 20 min after 50 mM EDC added to 100 mM propionic acid in MES buffer (200 mM, pH 6, 21 ^{o}C), injection volume: 0.5 µL. Compounds involved in the reaction network are propionic acid, benzylamine, N-acylurea, guanidine, and benzylamide. * Stands for the impurity from benzylamine solution.

We integrated peaks of guanidine, N-acylurea, and benzylamide to calculate the concentration of fuel, N-acylurea, and intermolecular anhydride to get the kinetics profile of the reaction cycle.

For the rest reaction cycles conducted under different conditions, we summarized their information on HPLC chromatograms in tables S7 and S8 (see above). The corresponding HPLC methods can be found in "**Materials and methods**".

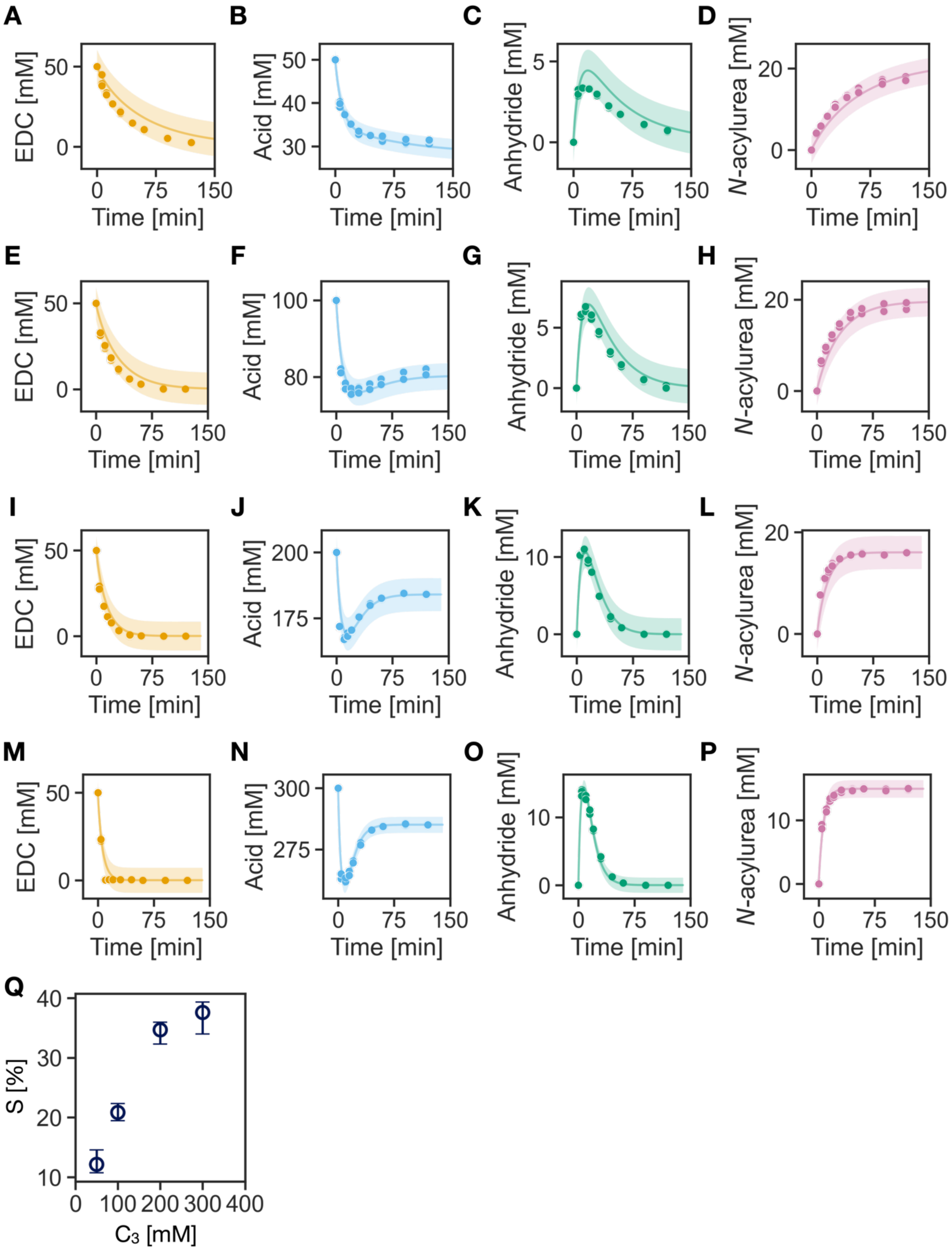

Figure S2. Kinetic profiles of reaction cycles fueled with 50 mM EDC in MES buffer (200 mM, pH 6 at 21°C) of (A-D) 50 mM propionic acid, **(E-H)** 100 mM propionic acid, **(I-L)** 200 mM propionic acid, **(M-P)** 300 mM propionic acid. Markers represent HPLC data and lines represent data from the kinetic model defined as 95 % confidence interval. The areas stand for the error area of our kinetic fitting. **(R)** Selectivity of the reaction cycle calculated by the kinetic model when different precursor concentration was used. All experiments were conducted in triplicates.

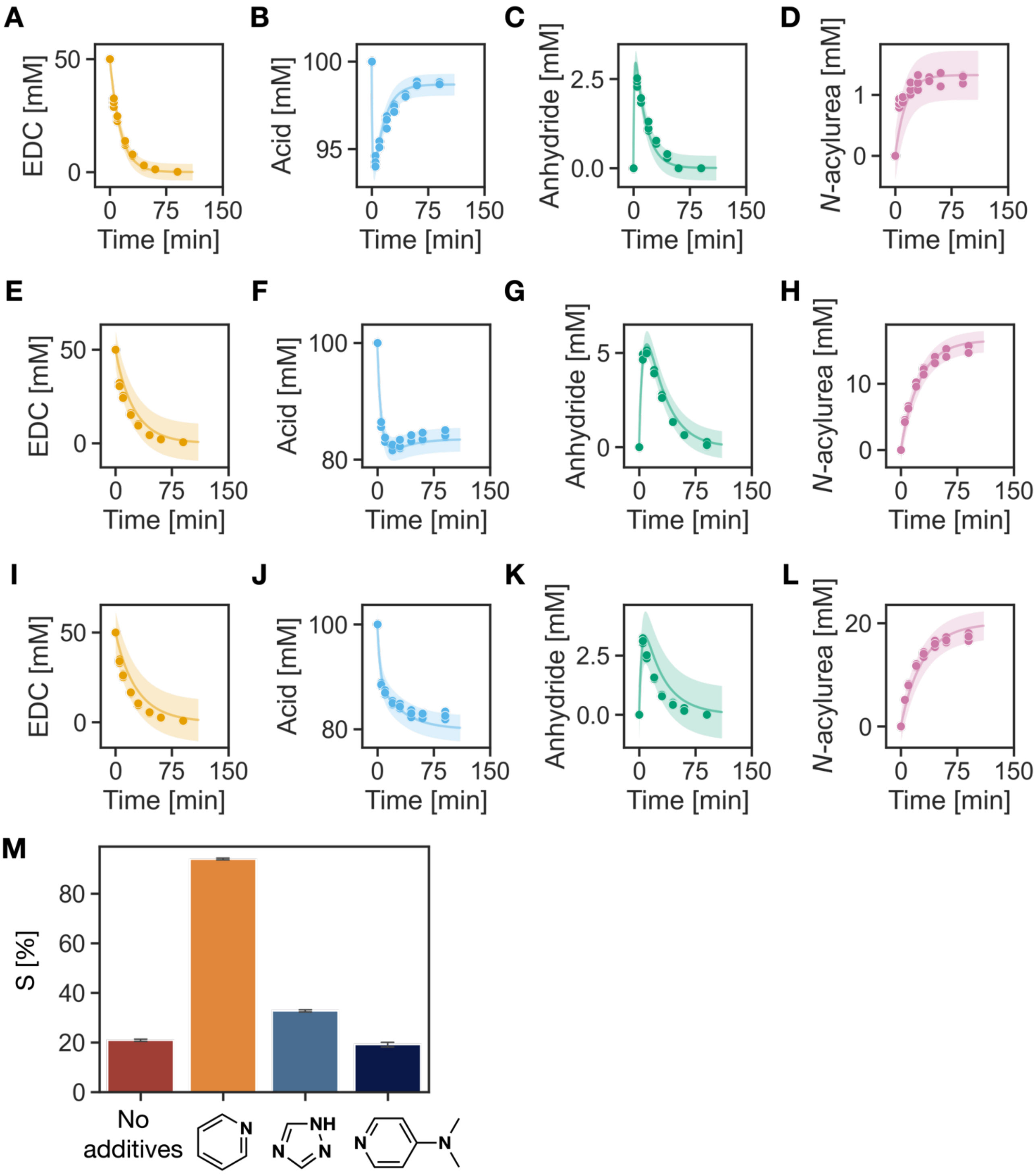

Figure S3. Kinetic profiles of reaction cycles of 100 mM propionic acid fueled with 50 mM EDC in MES buffer (200 mM, pH 6 at 21°C) with (A-D) 10 mM pyridine, **(E-H)** 10 mM triazole, **(I-L)** 10 mM DMAP. Markers represent HPLC data and lines represent data from the kinetic model defined as 95 % confidence interval. The areas stand for the error area of our kinetic fitting. **(M)** Selectivity of the reaction cycle calculated by the kinetic model when different additives were used. All experiments were conducted in triplicates.

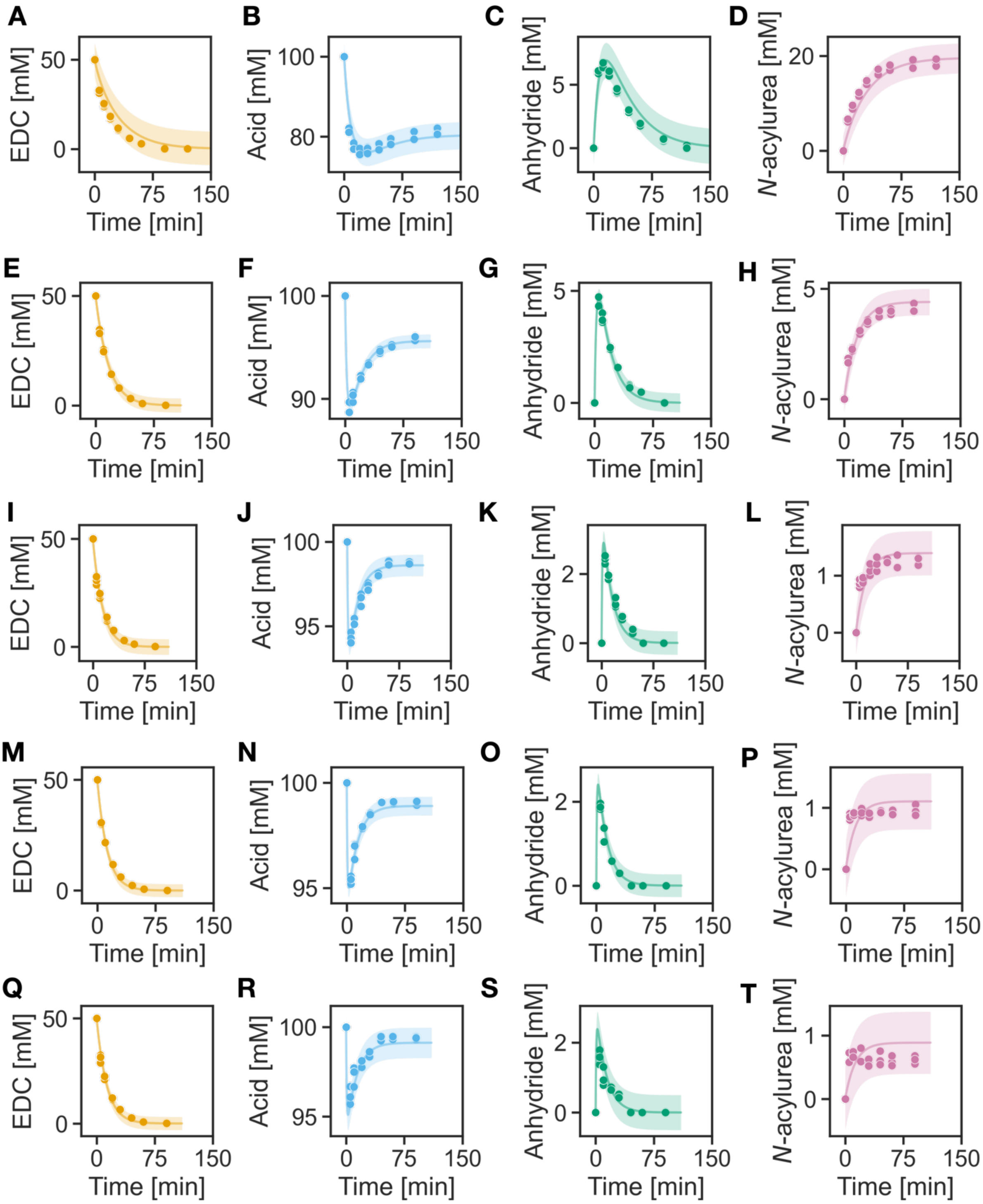

Figure S4. **Kinetic profiles of reaction cycles of 100 mM propionic acid fueled with 50 mM EDC in MES buffer (200 mM, pH 6 at 21°C) with (A-D)** 0 mM pyridine, **(E-H)** 5 mM pyridine, **(I-L)** 10 mM pyridine, **(M-P)** 20 mM pyridine, **(Q-T)** 35 mM pyridine. Markers represent HPLC data and lines represent data from the kinetic model. The areas stand for the error area of our kinetic fitting defined as 95 % confidence interval. All experiments were conducted in triplicates.

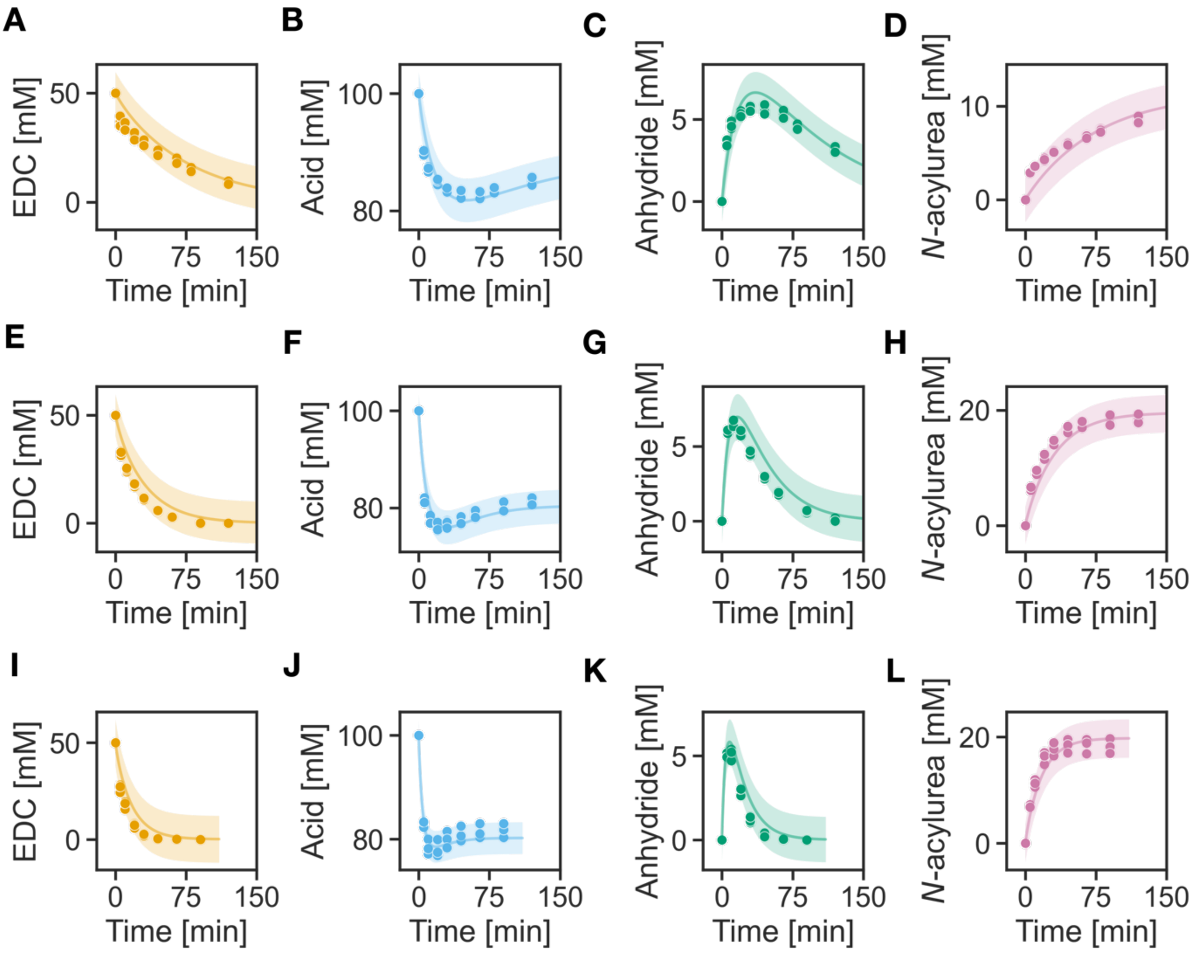

Figure S5. Kinetic profiles of reaction cycles of 100 mM propionic acid fueled with 50 mM EDC in MES buffer (200 mM, pH 6) at (A-D) 5 ^{o}C, **(E-H)** 21 ^{o}C, **(I-L)** 35 ^{o}C. Markers represent HPLC data and lines represent data from the kinetic model. The areas stand for the error area of our kinetic fitting defined as 95 % confidence interval. All experiments were conducted in triplicates.

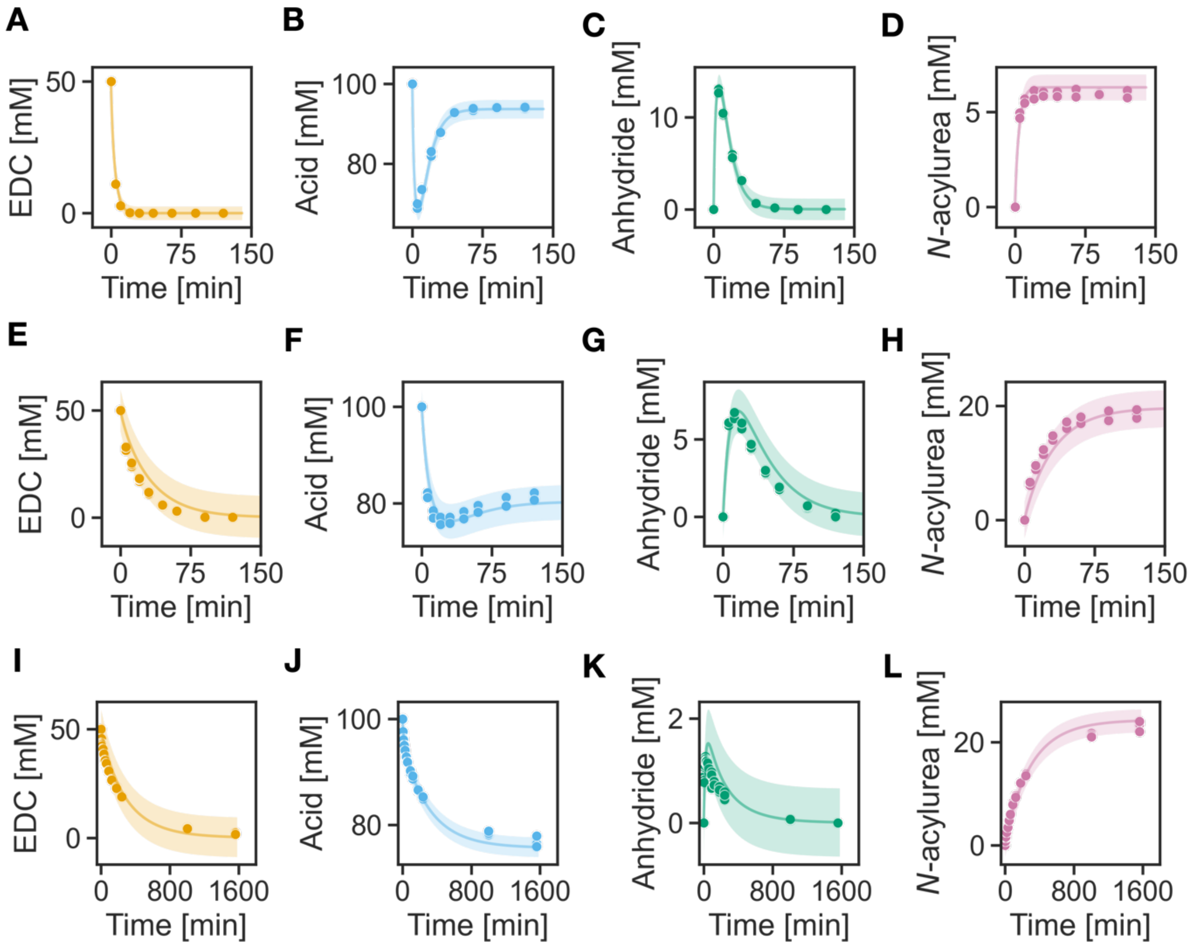

Figure S6. Kinetic profiles of reaction cycles of 100 mM propionic acid fueled with 50 mM EDC in MES buffer (200 mM, at 21 °C) at pH (A-D) 5, **(E-H)** 6, **(I-L)** 7. Markers represent HPLC data and lines represent data from the kinetic model. The areas stand for the error area of our kinetic fitting defined as 95 % confidence interval. All experiments were conducted in triplicates.

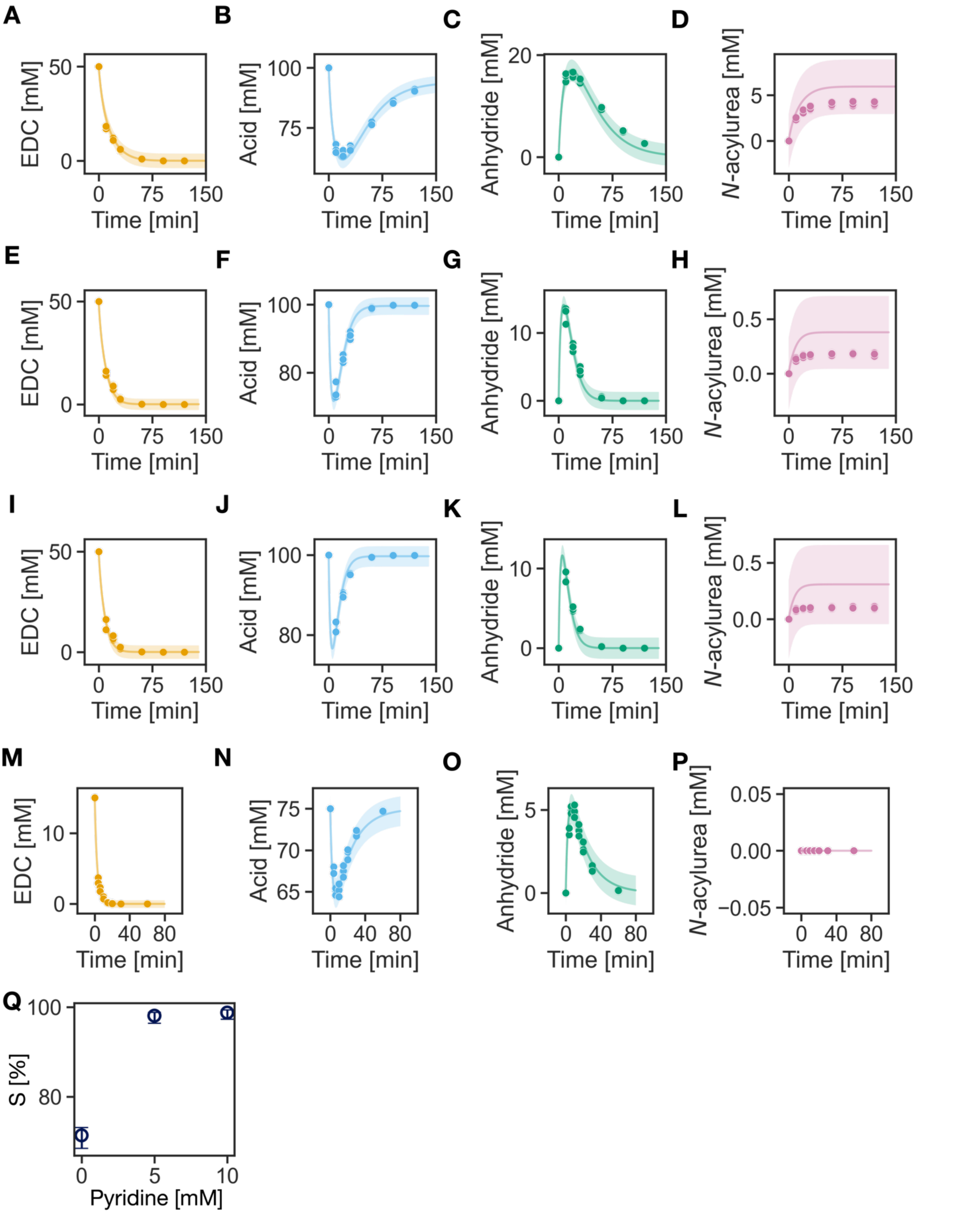

Figure S7. **Kinetic profiles of reaction cycles of 100 mM propionic acid fueled with 50 mM EDC in MES buffer (200 mM, pH 5 at 5 ^{o}C) with (A-D)** 0 mM pyridine, **(E-H)** 5 mM pyridine, **(I-L)** 10 mM pyridine. **(Q)** Selectivity of the reaction cycle under the above conditions calculated by the kinetic model. **(M-P) Kinetic profiles of 75 mM propionic acid with 1.5 mM pyridine fueled with 15 mM DIC in MES buffer (200 mM, pH 5 at 5 ^{o}C)**. Markers represent HPLC data and lines represent data from the kinetic model. The areas stand for the error area of our kinetic fitting defined as 95 % confidence interval. All experiments were conducted in triplicates.

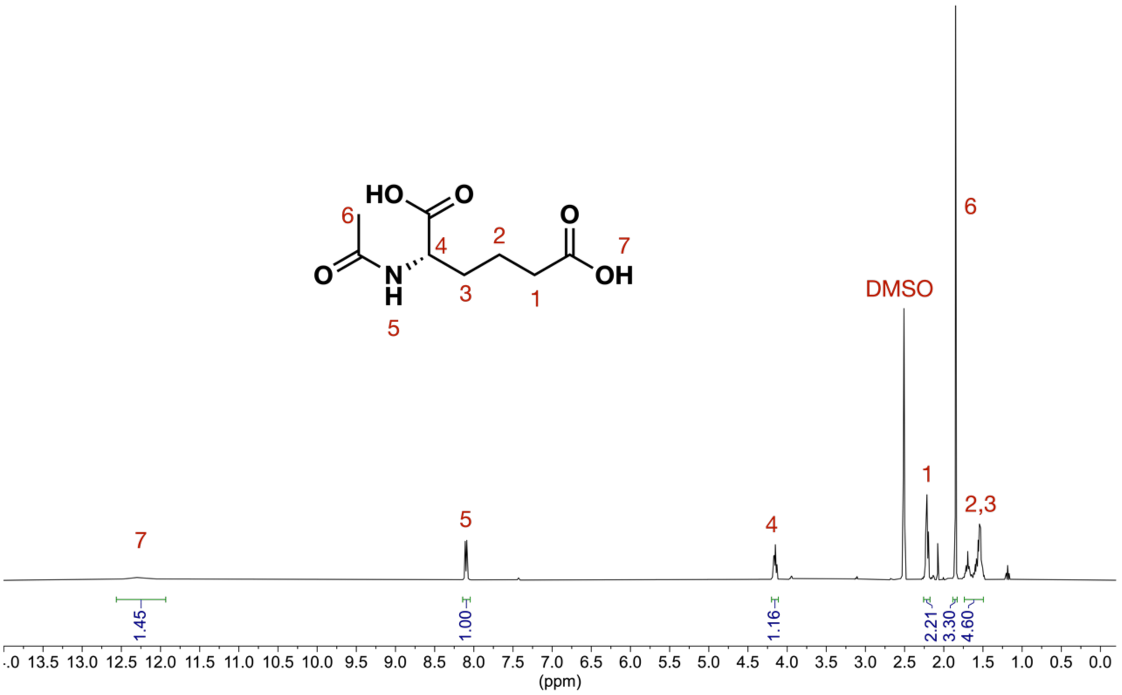

Figure S8. ^{1}H NMR spectrum (400 MHz, DMSO) of N-acetyl-L-2-aminoadipic acid. δ 12.29 (s, 1H), 8.10 (d, J = 7.8 Hz, 1H), 4.16 (td, J = 8.1, 5.0 Hz, 1H), 2.26 – 2.18 (m, 2H), 1.85 (s, 3H), 1.74 – 1.49 (m, 4H). Peak at δ 2.08 is the solvent ACN.

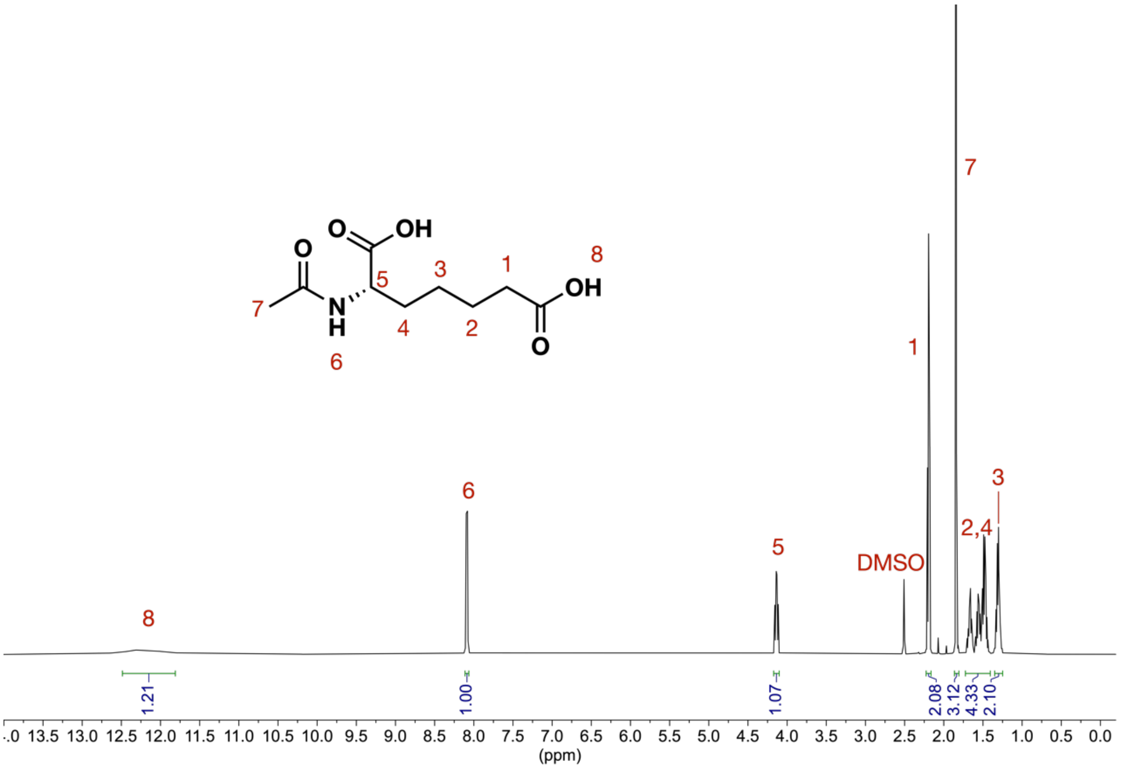

Figure S9. ^{1}H NMR spectrum (500 MHz, DMSO) of N-acetyl-L-2-aminoheptanedioic acid. δ 12.21 (s, 1H), 8.09 (d, J = 7.8 Hz, 1H), 4.14 (ddd, J = 8.9, 7.7, 4.9 Hz, 1H), 2.19 (t, J = 7.4 Hz, 2H), 1.84 (s, 3H), 1.72 – 1.41 (m, 4H), 1.35 – 1.25 (m, 2H).

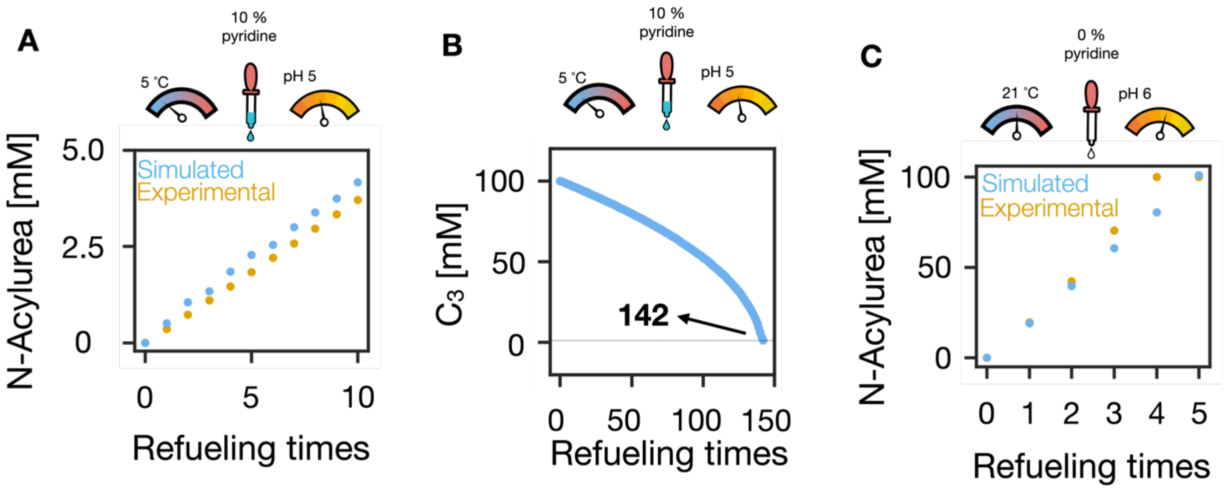

Figure S10: Refueling experiments of 100 mM propionic acid (C_3) fueled with 50 mM EDC in MES buffer under (A) our optimal condition (pH 5, 5°C, 10 % pyridine)**, (B**) the refueling times before the catalyst (C_3) concentration falls below 1 % simulated by our kinetic model and **(C)** the standard condition (pH 6, 21°C, 0 pyridine)**.**

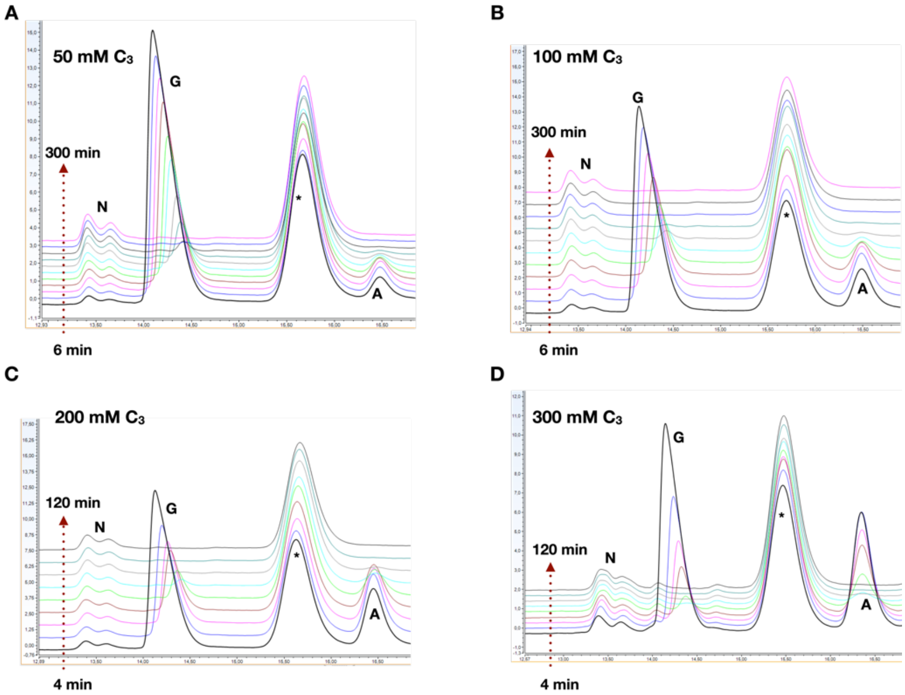

Figure S11: Representative HPLC chromatograms of reaction cycles fueled with 50 mM EDC in MES buffer (200 mM, pH 6 at 21°C) of (A) 50 mM propionic acid (C_3), **(B)** 100 mM C_3, **(C)** 200 mM C_3, **(D)** 300 mM C_3 over time at 254 nm (injection volume: 0.5 µL). Peaks from left to right are N-acylurea (N), guanidine (G), impurity from benzylamine (*) and benzylamide (A), respectively. Time traces from bottom to top are 6, 12, 20, 30, 45, 60, 90, 120, 180, 240, 300 min for 50 and 100 mM C_3. Time traces from bottom to top are 4, 10, 15, 20, 30, 45, 60, 90, 120 min for 200 and 300 mM C_3.

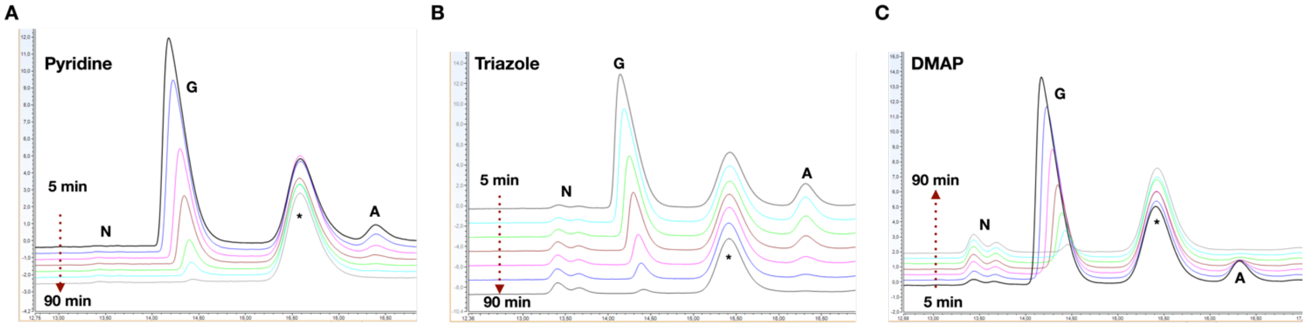

Figure S12: Representative HPLC chromatograms of reaction cycles of 100 mM propionic acid fueled with 50 mM EDC in MES buffer (200 mM, pH 6 at 21°C) with (A) 10 mM pyridine, **(B)** 10 mM triazole, **(C)** 10 mM DMAP over time at 254 nm (injection volume: 0.5 µL). Peaks from left to right are N-acylurea (N), guanidine (G), impurity from benzylamine (*) and benzylamide (A), respectively. Time traces from top to bottom are 5, 15, 20, 30, 45, 60, 90 min for pyridine. Time traces from top to bottom are 5, 10, 20, 30, 45, 60, 90 min for triazole. Time traces from bottom to top are 5, 10, 20, 30, 45, 60, 90 min for DMAP.

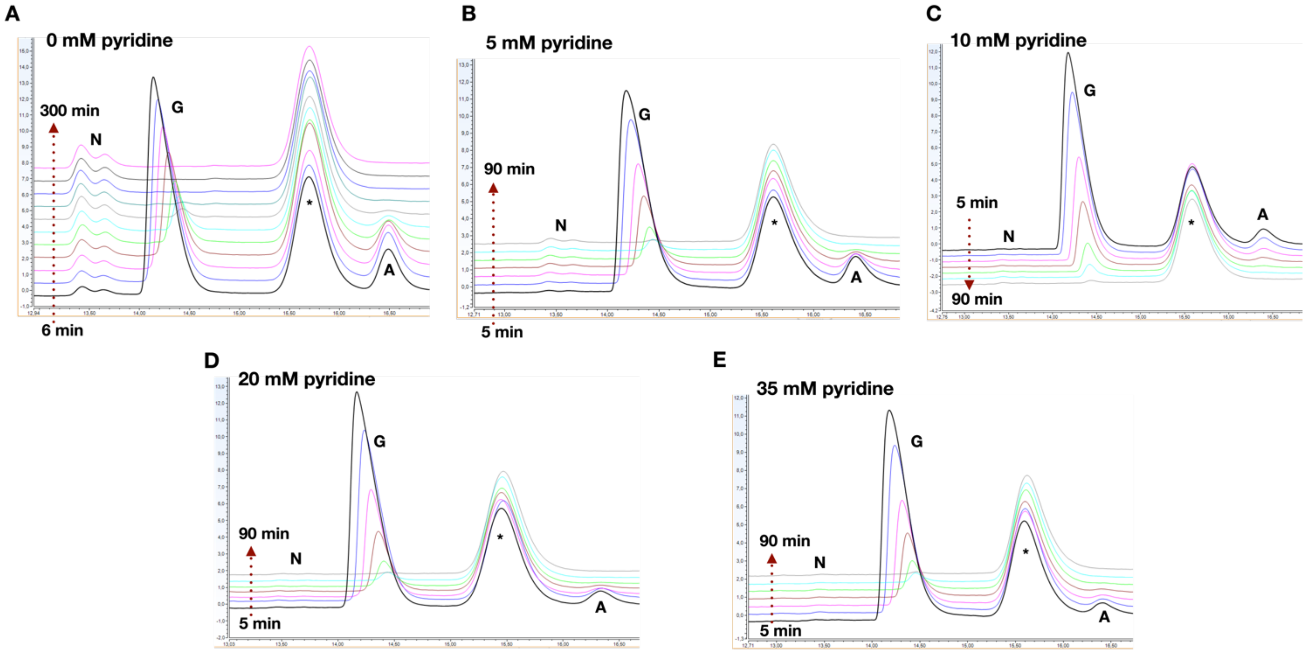

Figure S13: Representative HPLC chromatograms of reaction cycles of 100 mM propionic acid fueled with 50 mM EDC in MES buffer (200 mM, pH 6 at 21°C) with (A) 0 mM pyridine, **(B)** 5 mM pyridine, **(C)** 10 mM pyridine, **(D)** 20 mM pyridine, **(E)** 35 mM pyridine over time at 254 nm (injection volume: 0.5 µL). Peaks from left to right are N-acylurea (N), guanidine (G), impurity from benzylamine (*) and benzylamide (A), respectively. Time traces from bottom to top are 6, 12, 20, 30, 45, 60, 90, 120, 180, 240, 300 min for 0 mM pyridine. Time traces from bottom to top are 5, 10, 20, 30, 45, 60, 90 min for 5 mM, 20mM and 35 mM pyridine. Time traces from top to bottom are 5, 15, 20, 30, 45, 60, 90 min for 10 mM pyridine.

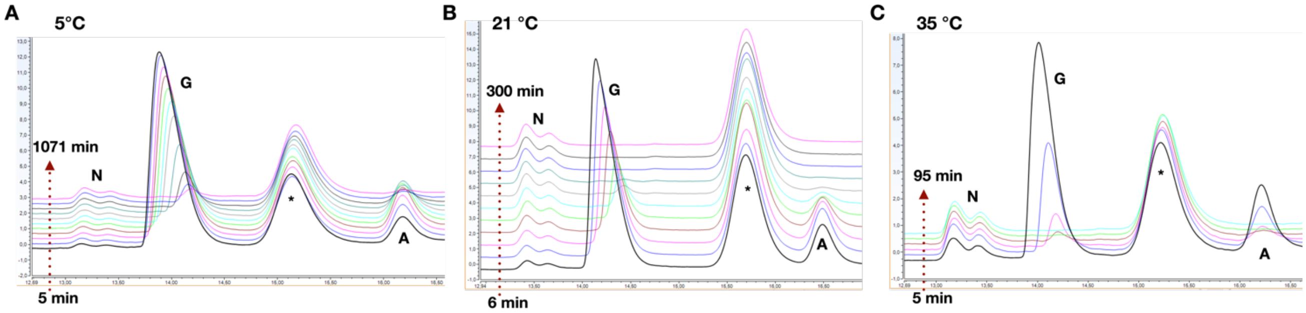

Figure S14: Representative HPLC chromatograms of reaction cycles of 100 mM propionic acid fueled with 50 mM EDC in MES buffer (200 mM, pH 6) at (A) 5 °C, **(B)** 21 °C, **(C)** 35 °C over time at 254 nm (injection volume: 0.5 µL). Peaks from left to right are N-acylurea (N), guanidine (G), impurity from benzylamine (*) and benzylamide (A), respectively. Time traces from bottom to top are 6, 12, 20, 30, 45, 60, 90, 120, 180, 240, 300 min for 21 °C. Time traces from bottom to top are 5, 10, 20, 30, 45, 65, 90, 120,180, 240, 300, 1071 min for 5 °C. Time traces from bottom to top are 5, 10, 20, 30, 45, 65, 95 min for 35 °C.

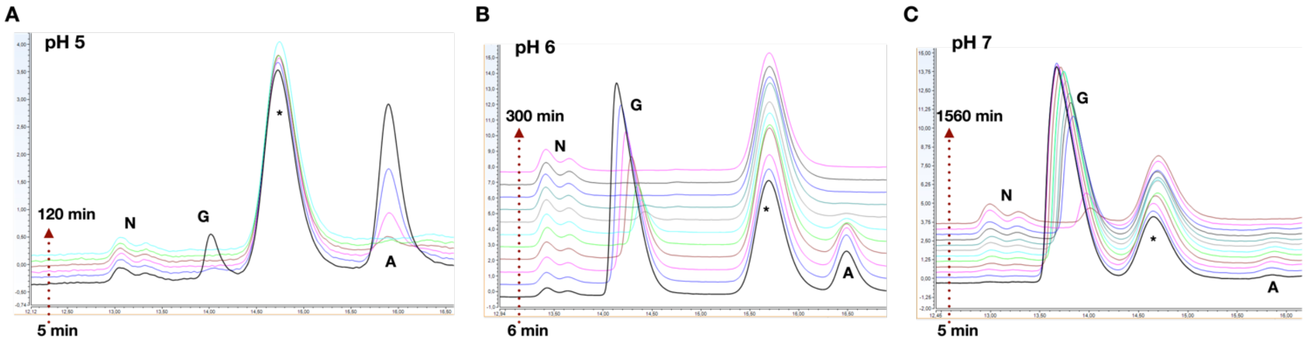

Figure S15: Representative HPLC chromatograms of reaction cycles of 100 mM propionic acid fueled with 50 mM EDC in MES buffer (200 mM, at 21 °C) at pH (A) 5, **(B)** 6, **(C)** 7 over time at 254 nm (injection volume: 0.5 µL). Peaks from left to right are N-acylurea (N), guanidine (G), impurity from benzylamine (*) and benzylamide (A), respectively. Time traces from bottom to top are 6, 12, 20, 30, 45, 60, 90, 120, 180, 240, 300 min for pH 6. Time traces from bottom to top are 5, 10, 20, 30, 45, 65, 90, 120 min for pH 5. Time traces from bottom to top are 5, 10, 20, 30, 45, 60, 90, 120, 180, 240, 1003, 1560 min for pH 7.

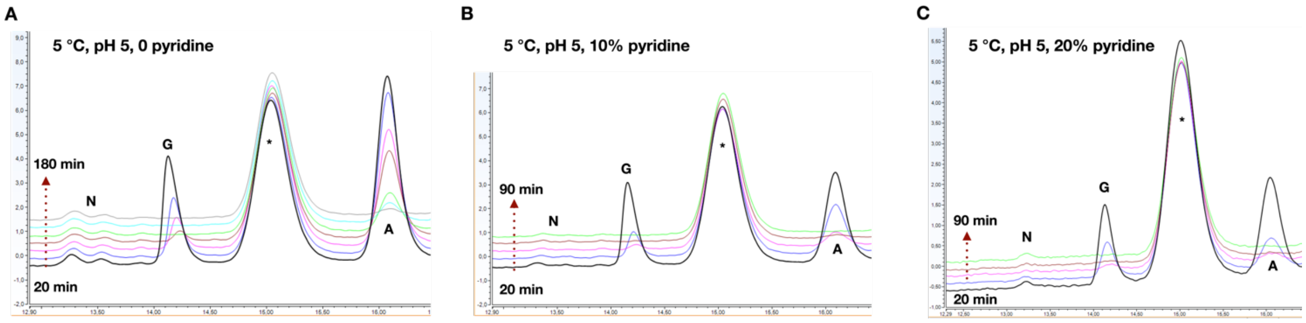

Figure S16: Representative HPLC chromatograms of reaction cycles of 100 mM propionic acid fueled with 50 mM EDC in MES buffer (200 mM, pH 5 at 5 °C) with (A) 0 mM pyridine, **(B)** 5 mM (10%) pyridine, **(C)** 10 mM (20%) pyridine over time at 254 nm (injection volume: 0.5 µL). Peaks from left to right are N-acylurea (N), guanidine (G), impurity from benzylamine (*) and benzylamide (A), respectively. Time traces from bottom to top are 20, 30, 45, 60, 90, 120, 180 min for 0 mM pyridine. Time traces from bottom to top are 20, 30, 45, 60, 90 min for 5 and 10 mM pyridine.

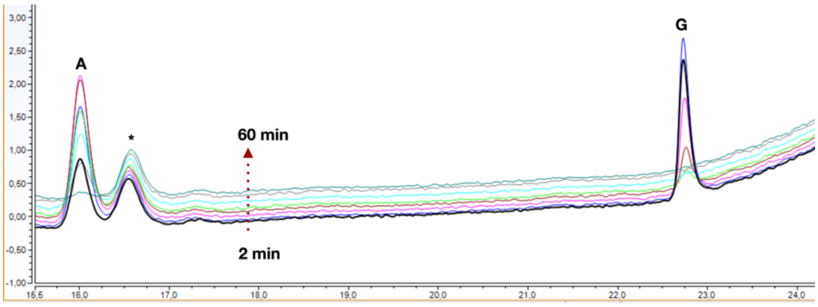

Figure S17: Representative HPLC chromatograms of reaction cycles of 75 mM propionic acid in MES buffer with 1.5 mM pyridine fueled with 15 mM DIC in MES buffer (200 mM, pH 5 at 5°C). Peaks from left to right are benzylamide (A), impurity from benzylamine (*) and guanidine (G), respectively. No N-acylurea was formed. Time traces from bottom to top are 2, 4, 6, 10, 15, 20, 30, 60 min.

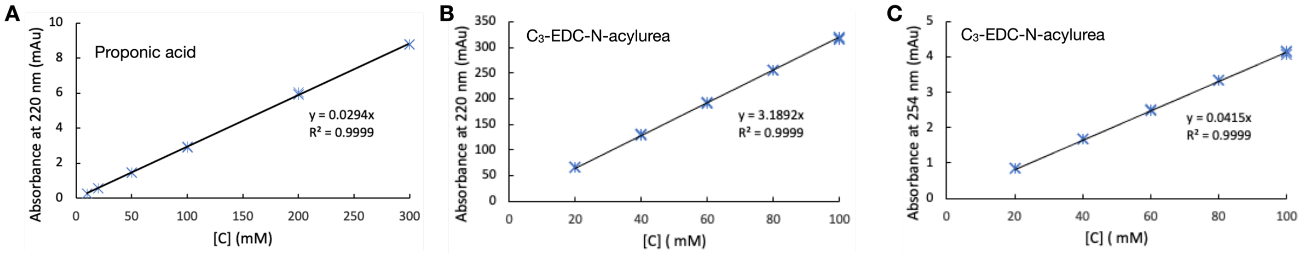

Figure S18: Calibration curves of 0.5 µL injection volume (A) propionic acid (220 nm), **(B)** N-acylurea of propionic acid when EDC is the fuel at 220 nm and **(C)** at 254 nm. Error bar: n = 3.

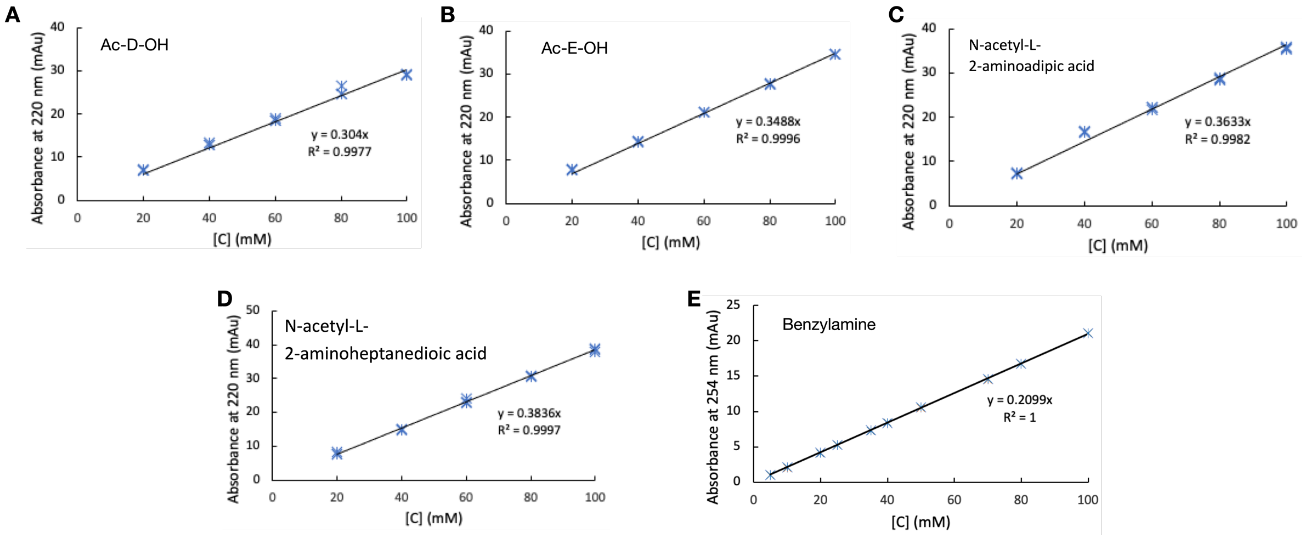

Figure S19: Calibration curves of 0.5 µL injection volume (A) Ac-D-OH (220 nm), **(B)** Ac-E-OH (220 nm), **(C)** N-acetyl-L-2-aminoadipic acid (220 nm), **(D)** N-acetyl-L-2-aminoheptanedioic acid (220 nm), **(E)** benzylamine (254 nm). Error bar: n = 3.

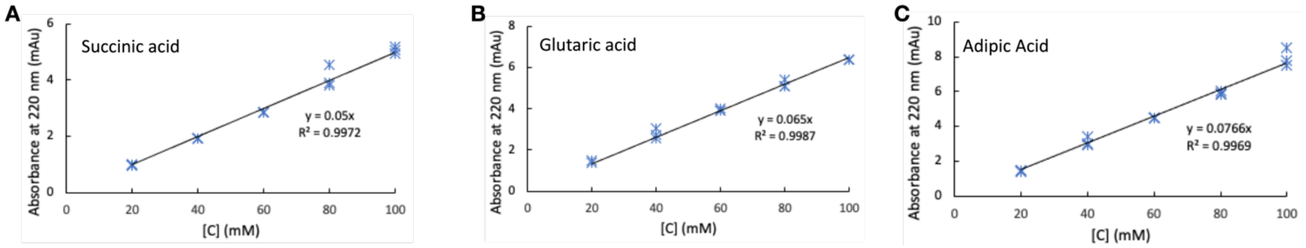

Figure S20: Calibration curves of 0.5 µL injection volume (A) Succinic acid (220 nm), **(B)** Glutaric acid (220 nm), **(C)** Adipic Acid (220 nm). Error bar: n = 3.

REFERENCE

1. A. V. Reddy and B. Ravindranath, Acetylation Under Ultrasonic Conditions: Convenient Preparation of N-Acetylamino Acids, *Synthetic Communications*, 2006, **22**, 257-264.
2. F. Schnitter and J. Boekhoven, A Method to Quench Carbodiimide-Fueled Self-Assembly, *ChemSystemsChem*, 2020, **3**, e2000037.
3. L. S. Kariyawasam and C. S. Hartley, Dissipative Assembly of Aqueous Carboxylic Acid Anhydrides Fueled by Carbodiimides, *J. Am. Chem. Soc.*, 2017, **139**, 11949-11955.
4. L. S. Kariyawasam, J. C. Kron, R. Jiang, A. J. Sommer and C. S. Hartley, Structure-Property Effects in the Generation of Transient Aqueous Benzoic Acid Anhydrides by Carbodiimide Fuels, *J. Org. Chem.*, 2020, **85**, 682-690.
